# Functional site plasticity in domain superfamilies^☆^

**DOI:** 10.1016/j.bbapap.2013.02.042

**Published:** 2013-05

**Authors:** Benoit H. Dessailly, Natalie L. Dawson, Kenji Mizuguchi, Christine A. Orengo

**Affiliations:** a National Institute of Biomedical Innovation, ZIP:567-0085, 7-6-8 Asagi Saito Ibaraki-City Osaka, Japan; b Institute of Structural and Molecular Biology, Division of Biosciences, University College London, Gower Street, London WC1E 6BT, UK

**Keywords:** Protein domain structure, Functional diversity, Structural diversity, Functional residues, Functional site diversity

## Abstract

We present, to our knowledge, the first quantitative analysis of functional site diversity in homologous domain superfamilies. Different types of functional sites are considered separately. Our results show that most diverse superfamilies are very plastic in terms of the spatial location of their functional sites. This is especially true for protein–protein interfaces. In contrast, we confirm that catalytic sites typically occupy only a very small number of topological locations. Small-ligand binding sites are more diverse than expected, although in a more limited manner than protein–protein interfaces. In spite of the observed diversity, our results also confirm the previously reported preferential location of functional sites. We identify a subset of homologous domain superfamilies where diversity is particularly extreme, and discuss possible reasons for such plasticity, i.e. structural diversity. Our results do not contradict previous reports of preferential co-location of sites among homologues, but rather point at the importance of not ignoring other sites, especially in large and diverse superfamilies. Data on sites exploited by different relatives, within each well annotated domain superfamily, has been made accessible from the CATH website in order to highlight versatile superfamilies or superfamilies with highly preferential sites. This information is valuable for system biology and knowledge of any constraints on protein interactions could help in understanding the dynamic control of networks in which these proteins participate. The novelty of our work lies in the comprehensive nature of the analysis – we have used a significantly larger dataset than previous studies – and the fact that in many superfamilies we show that different parts of the domain surface are exploited by different relatives for ligand/protein interactions, particularly in superfamilies which are diverse in sequence and structure, an observation not previously reported on such a large scale. This article is part of a Special Issue entitled: The emerging dynamic view of proteins: Protein plasticity in allostery, evolution and self-assembly.

## Highlights

• Most diverse domain superfamilies have very diverse functional site locations.
• Catalytic sites are found in a small, restricted number of topological positions.
• Location of small-ligand binding sites is more diverse than expected.
• Protein–protein interfaces display the most flexibility in functional site locations.

## 1 Introduction

Functional sites are sets of residues that are directly involved in the function of proteins. Those generally include binding sites to other molecules, and catalytic sites, a subset thereof, which are involved in the actual catalytic mechanism of enzymes.

Knowing which residues in a protein are important for its function is important for a number of reasons. For example, it makes it easier to modify the function of that protein by site-directed mutagenesis. It can also help identify potential interacting partners for the protein.

Because of their importance, much effort has been put into the development of computational methods to predict the location of functional sites (see for example [1]). One prevalent approach to predicting the location of a functional site in a query protein, is to transfer known functional site data from homologous proteins. The assumption with this type of approach is that homologous proteins should have functional sites at equivalent locations.

Generally, such homology-based approaches tend to become more reliable as the sequence similarity between proteins increases [2]. However, a number of studies have explored the possibility of transferring functional site information between remotely related homologues [3–5]. Some studies have even suggested that functional sites could be transferred with some success between proteins that share structural similarities but no evidence of homology [6,7].

Proteins are made up of domains, i.e. units of protein evolution that have well-defined structures [8]. Several resources have been established to group such domains by homology into so-called “domain superfamilies” [9,10]. It is generally well accepted that such homologous domains share some level of functional similarity, and that intermolecular interactions and the functional sites mediating them can often be inherited between them [6,11].

In-depth analysis of protein domain superfamilies has shown that related domains may often adopt diverse structures, and perform a variety of functions [12]. This is particularly true for a subset of superfamilies that are very diverse, and that are populated by large numbers of domains [13,14,8].

The observation that structural diversity is often more pronounced in regions directly involved in function [15,16] points at the possibility that functional sites may vary significantly between related protein domains in those large and diverse superfamilies. Over the years, a number of studies have shown that the underlying assumption that homologous proteins have their sites in similar locations, may not always hold true.

For example, it was shown that similar domain pairs tend to interact in the same way when sequence identity is 30–40% or higher, but that more remote similarities (at the fold level for example) are rarely associated with a similarity in interaction [2].

Previous studies have attempted to look at how diverse the functional sites of different types are between sets of homologous proteins, genes or domains. For example, it was reported that domains within SCOP families (the family level in SCOP groups together domains that are clearly evolutionarily related, generally with pairwise sequence identities of 30% or greater), generally have their binding sites in similar locations [17]. As part of a review on challenges to predict macromolecular interactions, Wass et al. succinctly reported a count of ligand-binding sites in SCOP superfamilies and described that most superfamilies have a small number of such sites, and that these sites tend to be found in most superfamily members [11].

However, an exhaustive and quantitative analysis of site location diversity for all types of functional sites among related protein domains at the superfamily level remains to be performed. That is the purpose of this study. We have compared the spatial location of different types of functional sites between related domains, in an exhaustive manner for all superfamilies. For that, we first select a representative in each superfamily of our dataset, and then map functional sites from each domain in the superfamily onto the representative. We consider different types of functional sites separately, and present results for each type. In an attempt to compensate for the lack of functional site data in the PDB, we then go on to perform the same analysis but this time exploiting a simple and intuitive protein functional site predictor, i.e. sequence conserved residues. We report that domains in large and diverse superfamilies, can have sites in very diverse locations, especially for protein–protein binding sites, and small ligand binding sites. We observe that large superfamilies with many domains are the most likely to have sites in different locations, especially if their domains are also structurally diverse. This is a novel observation not previously reported in the literature and has been obtained by performing a more comprehensive analysis than earlier studies. It is valuable in drawing attention to superfamilies where more caution may need to be employed when inheriting functional site data between relatives.

Our results simply reflect what is currently observed in the PDB when all known site data for a given superfamily is sampled. They are purely empirical and not intended to provide probabilities for particular binding sites.

Our functional site mappings are made publicly available for all superfamilies via the CATH website.

## 2 Methods

### 2.1 Definition of homologous protein domains

In our study, protein domains are considered homologous if they are part of the same CATH superfamily [9]. Within CATH superfamilies, domains are further clustered at 60% sequence identity.

Some superfamilies have been shown to be very large and very diverse [14]. These 60% sequence identity clusters (also called s60 clusters) are useful to quantify diversity in superfamilies. Indeed, 60% sequence identity has been shown in several studies to be a reasonable threshold for grouping domains with similar functions [18]. Therefore, the number of clusters at 60% sequence identity provides an approximation of the number of diverse functions in the superfamily.

We use version 3.5 of the CATH database, which consists of 2626 superfamilies. Since we are focusing on functional site diversity, we exclude all superfamilies consisting of a single 60% cluster, or with no functional site data. Our final dataset consists of 1456 superfamilies.

### 2.2 Definition of functional families

Functional families have recently been introduced to the CATH-Gene3D database [19]. CATH superfamily sequence data are clustered into functional families, using two related approaches (GeMMA [20] and Domain Family eXploration program [21]), which consist of relatives likely to have the same function. The GeMMA algorithm identifies functional families using a hierarchical agglomerative clustering algorithm to produce a tree of clusters built from the leaf nodes to the root node. This iterative approach first clusters close homologues, i.e. sequences with at least 90% sequence identity, using the program CD-HIT [22]. For each of these clusters, multiple sequence alignments (MSAs) are constructed using MAFFT [23]. In the second iteration, pairs of MSAs are compared using the Comparison of Multiple Protein Alignments with Assessment of Statistical Significance (COMPASS) set of tools [24]. COMPASS takes two MSAs as input and from these builds two PSSM profiles for comparison purposes. It then calculates the similarity of all profile pairs, and the alignments with the highest similarity are merged. This continues until one cluster remains. The final tree of clusters is partitioned by cutting the tree at a generic threshold. This approach is referred to as the ‘unsupervised’ method and produces ‘fine’ functional families (FineFams) [20]. The FineFams have been benchmarked against the Structure Function Linkage Database (SFLD) [25].

A modified version of the functional families has since been developed using a ‘supervised’ protocol [21]. This approach (DFX) detects and accounts for functional ‘chaining’ within the tree of clusters. ‘Chaining’ refers to instances of protein domain sequences in a superfamily that cluster in an unexpected way. In DFX, GO annotation data is used to ensure functional coherence in each functional family, and clusters are only merged if they contain coherent GO terms. However, in some superfamilies the sequence similarity reflected in the COMPASS scores appears to contradict GO term similarity so that domain relatives apparently having different functions are preferentially merged in the hierarchy. This phenomenon usually arises because in these superfamilies domains have a generic functional role that remains unchanged despite the different functional contexts (reflected in different GO terms for their parent proteins) in which the relatives appear. The DFX method is described in more detail in Rentzsch et al. [21] and tends to produce ‘coarser’ functional families (FunFams), in which domain relatives in a cluster are likely to have similar functional roles but the proteins in which they are found may have different overall functions.

This is illustrated by the fact that relatives in FunFams tend to superpose with higher RMSD than relatives within FineFams (see Supplementary material Fig. A.10), suggesting that since there is a known correlation between structural and functional diversity [14], FineFam relatives are closer in function.

As functional families carry out the same general function, each sequence member is expected to contain the same residues required for that function. Such sites will therefore be highly conserved throughout a functional family.

Using these functional families allows us to study these highly conserved functional residues and observe how such residues compare between functional families within a superfamily, thereby giving an impression of how functional sites evolve within a superfamily.

### 2.3 Definition of functional sites

Catalytic residues are defined as manually curated residues from the Catalytic Site Atlas (CSA) [26].

Binding sites are defined using the NCBI Inferred Biomolecular Interaction Server (IBIS) [27]. IBIS provides information about binding sites for all types of ligands, including other proteins, nucleic acids, small organic compounds, peptides and ions. Binding sites for these different types of ligands were considered separately throughout this work. We also used quality filters provided by IBIS when selecting binding site data. In particular, we ignore any ligands that are not considered biologically relevant by IBIS. And we also exclude interfaces that are not confirmed by PISA [28]. IBIS functional residues inferred by homology are not considered here. Finally, in our work, we group together small organic compounds, peptides and ions in a common category that we refer to as “small ligands”.

Some proteins have several structures in the PDB [29]. Some structures may contain ligands and others will not. To ensure maximal coverage, we always consider all structures of a given protein when collecting its functional site residues.

### 2.4 Mapping functional sites between members of the superfamily

**Fig. 1:**
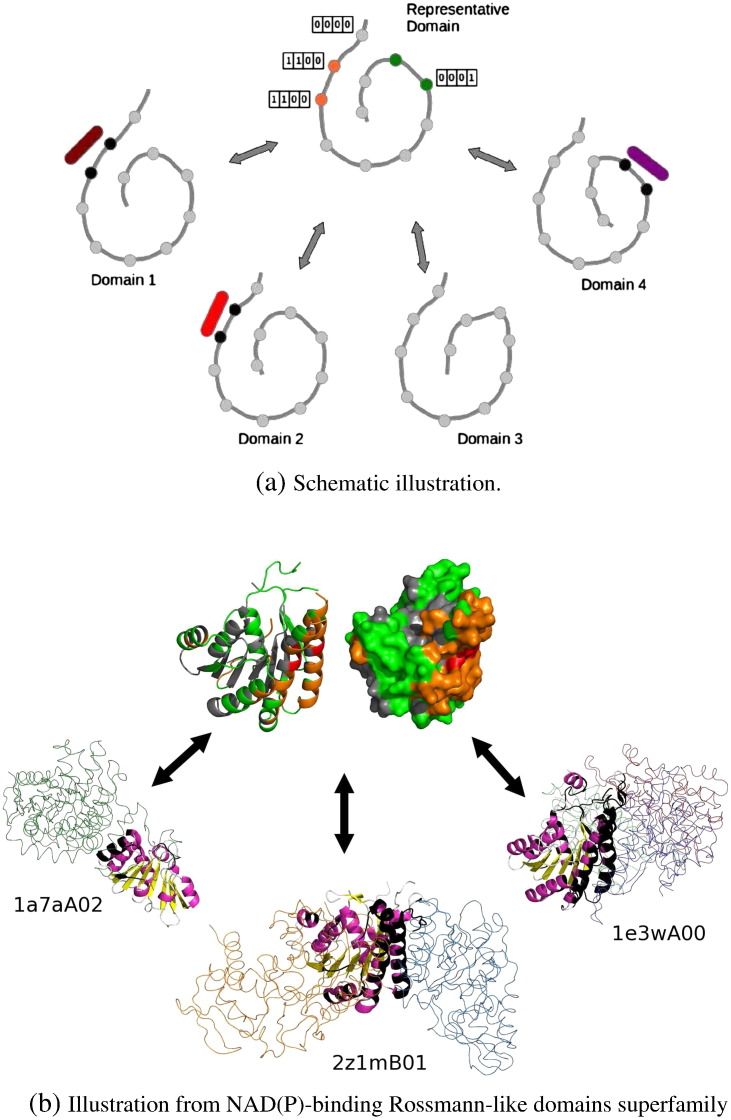
Mapping protocol. Fig. 1a illustrates the protocol schematically. All domains in a superfamily (domains 1 to 4) are structurally aligned to a superfamily representative. Domains are represented as a dark grey backbone, and individual residues are represented as beads along the backbone. Ligands are represented as purple, red and magenta ellipsoids that bind to domains 1, 2 and 4, respectively. Binding residues in these domains are coloured in black. Binding residues from the individual domains are then mapped to the representative, and the frequency with which representative residues map to binding residues is recorded. In this example, the residues in orange on the representative map to binding residues in two domains (domains 1 and 2), whereas the residues in green map to binding residues in only one domain (domain 4). The vectors next to some of the positions of the representative summarise the list of superfamily domains where equivalent residues are involved in binding. Fig. 1b illustrates the protocol with real protein-protein interface data from domains in the NAD(P)-binding Rossmann-like superfamily. Three individual domains from the superfamily are represented in complex with their protein partners at the bottom, and their interface residues are mapped on the representative at the top. The domains of interest are shown in cartoon whereas the partner chains are represented as thin linear chains. The representative is shown both in cartoon and surface representation. Binding residues in the individual domains are coloured black. Residues on the representative are coloured grey, green, orange or red depending on the number of individual domains that have a binding residue at that position (0, 1, 2 or 3).

Our approach for comparing the three-dimensional location of functional sites across members of the superfamily consists of three steps. The mapping protocol is illustrated in Fig. 1.

First, we select a representative for each superfamily. The idea is to select the superfamily member that is the best structural representative of the superfamily. In CATH, all 60% sequence identity clusters have a pre-defined representative. We structurally align all these representatives pair-wise against one another using the program SSAP [30]. The superfamily representative is then selected as the domain that has the highest cumulative structural similarity SSAP score to all other sequence clusters in the superfamily. The superfamily representative is therefore supposed to be the domain that is most structurally similar to all other domains in the superfamily. This notably minimizes the risks of misalignments between the representative and other superfamily members.

The SSAP algorithm has been benchmarked using manually validated structure comparisons and has been shown to perform well in identifying equivalent regions between homologous domains [31]. Even very remote homologues share a conserved structural core, usually comprising at least 50% of residues, which can be well aligned between them (see Supplementary material Fig. A.11 for an example of the superposition of two very diverse relatives (sequence identity 6%, RMSD 14 Å), showing the conserved core). Multiple pair-wise superpositions of homologues within each CATH superfamily, revealing the structurally conserved core, can be seen on the CATH website.

Once the representative is chosen, we then structurally align it against all superfamily members' domains (i.e. not just the sequence cluster representatives). This provides us with a structure-based residue-mapping between all superfamily members and the representative. As before, we use SSAP for the structural alignments.

Finally, we map the functional site residues in individual superfamily members onto the superfamily representative. Therefore for each position in the representative, we know whether it maps to functional residues in any superfamily members, and what these superfamily members are. If a functional residue from another domain in the superfamily maps to a gap in the structural alignment with the representative domain, that residue is ignored in the rest of the analysis. The frequency at which individual residues on the representative domain have their equivalent residues in other domains involved in functional interactions can be counted (see Fig. 1).

We decided not to limit our analysis to surface positions, for several reasons. First, there is no universally accepted definition of surface residue. Secondly, it is common for residues that should be considered buried by most standards, (e.g. relative surface accessibility lower than 0.05) to be involved in function. For example, it was reported that catalytic sites have low relative solvent accessibilities, and up to 5% of them are fully buried [32]. Also, a position that is buried in one structure may be surface-accessible in another structure of the same protein due to conformational changes. Finally, functional residues used in this work are obtained from the CSA and IBIS resources, and were originally defined as functional based on a careful analysis of the literature, or using protein-ligand complexes of known structure in the PDB. In both resources, buried residues may be considered functional, and in order not to lose any data, we chose not to include extra constraints of surface accessibility.

In addition to performing a mapping of sites to a superfamily representative, we have used the same strategy to map sites for a functional family (FineFam) to the FineFam representative, in order to examine site coverage for a more functionally coherent grouping of relatives.

### 2.5 Conservation analysis

Sequences in functional families are aligned using MAFFT [33] and conservation scores for each position in the alignments are computed using Scorecons [34]. A conservation score threshold of 0.7 was empirically chosen to define conserved residues, based on previous studies in our group and comparison with known functional residues in a subset of alignments.

### 2.6 Computing overlap between conserved and functional residues

Residue enrichment analysis has been used to assess the purity of different types of functional families (i.e. FunFams or FineFams). Functional families ideally contain sequences that code for a protein with the same molecular function, and therefore the same functional residues, e.g. catalytic residues, are expected to feature in all of the sequences throughout the functional family as they will be highly conserved.

We have performed enrichment tests, for both FunFam and FineFam functional families, to ensure that conserved residues are enriched in functional residues compared to the background dataset of residues, i.e. all residues in the proteins. For this, we followed the procedure explained in a previous study [35]. The idea is to compute the proportion of conserved residues that are also functional, Pc, and the proportion of all residues that are also functional, Pa. The enrichment E, is equal to the difference of Pc − Pa. Enrichment values are then averaged over superfamilies, and a Wilcoxon Rank-Sum test [36] is performed over the set of enrichments to check whether Pc values tend to be significantly larger than Pa values. Wilcoxon Rank-Sum tests were performed using the function wilcox.test in R [37].

Enrichment scores were also calculated for FunFam and FineFam alignments stripped of partial sequence fragments. These fragments are expected to affect the quality of the alignments by introducing numerous gaps between fragments and full-length protein domain sequences. Sequences with a length less than 80% of the corresponding functional family average sequence length were removed. The remaining functional family sequences were aligned using MAFFT and residue conservation scores were calculated using Scorecons. Enrichment scores were then calculated as previously described.

### 2.7 Hub analysis

This section and the following one refer to analyses that we performed to help the interpretation of our results on functional site diversity.

Data about hubs (from protein interaction networks) was collected by integrating interaction data from IntAct [38], Mint [39], BioGrid [40], DIP [41], HPRD [42], Reactome [43] and Virus-Host-Net [44]. For all proteins in the human genome (Ensembl [45], release 62), a network of interactions was built by filtering to include only physical interactions. CATH domains were then mapped to their parent proteins in the network, and the number of physical interactions for each parent protein was mapped back to the domain. Hub superfamilies were defined as any superfamily in which at least one member is involved in at least 10 interactions. Other cut-offs were considered and did not result in significant changes of the overall distribution of hub superfamilies (data not shown).

### 2.8 Structural diversity analysis

Structural diversity within superfamilies can be described quantitatively by clustering domains within them according to structural similarity, and then counting the number of structural clusters in the superfamily at a given cut-off of structural similarity.

Structural similarity was quantified using a normalised RMSD score, which consists of the RMSD multiplied by the length of the largest domain in the pair, and then divided by the number of aligned residue pairs between the 2 domains (see [46] for more details).

The number of clusters then provides a global measure of structural diversity within superfamilies that can be used to compare different superfamilies. The structural clusters are identified as described in the CATH database, using a normalised RMSD cutoff of 9.0 Å to define the clusters. We then define structurally diverse superfamilies as those superfamilies that have at least 2 structural clusters. We also repeated this analysis using a cutoff of 5.0 Å rather than 9.0 Å.

## 3 Results

### 3.1 Functional site coverage

In order to evaluate the diversity of functional site spatial locations amongst members of the same superfamily, we used a strategy whereby a superfamily representative is chosen, and sites from all members of the superfamily are then mapped to it via pairwise structural alignments.

One simple measure of functional site diversity across a superfamily is the coverage of the representative by functional sites, i.e. the number of positions on the representative that map to a functional site of a given type (e.g. protein–protein binding sites), divided by the total number of positions in the representative. If the representative domain is not too small, high coverage will generally mean that functional sites from superfamily members can occur at different spatial locations, that when mapped onto the representative, cover most of its positions.

Two confounding factors must be taken into account with this measure of diversity. First, if the representative domain is very small, it is more likely to have a very high coverage. We avoid that problem by considering only superfamilies where the representative has at least 100 residues. Of the 1456 superfamilies in our initial dataset, 908 have a large enough representative.

Secondly, high coverage may in some cases be due to a single superfamily member. Indeed, if a single domain in the superfamily has most of its residues involved in interactions with partner molecules, the coverage on the representative may be very high. But in such cases, high coverage is not indicative of diversity of functional sites between superfamily members. Although such cases are arguably of interest, they are not the object of the present analysis, and we have kept them out for clarity. In practice, any superfamily where one domain contributes more than 50% coverage of the representative is ignored from our analysis. However, results obtained when including these superfamilies can be seen in Supplementary material Fig. A.12.

The number of superfamilies that pass this second filter depends on the type of site being considered. Table 1 shows, for each type of functional site, the number of superfamilies left in the dataset after all filters have been applied.

**Fig. 2:**
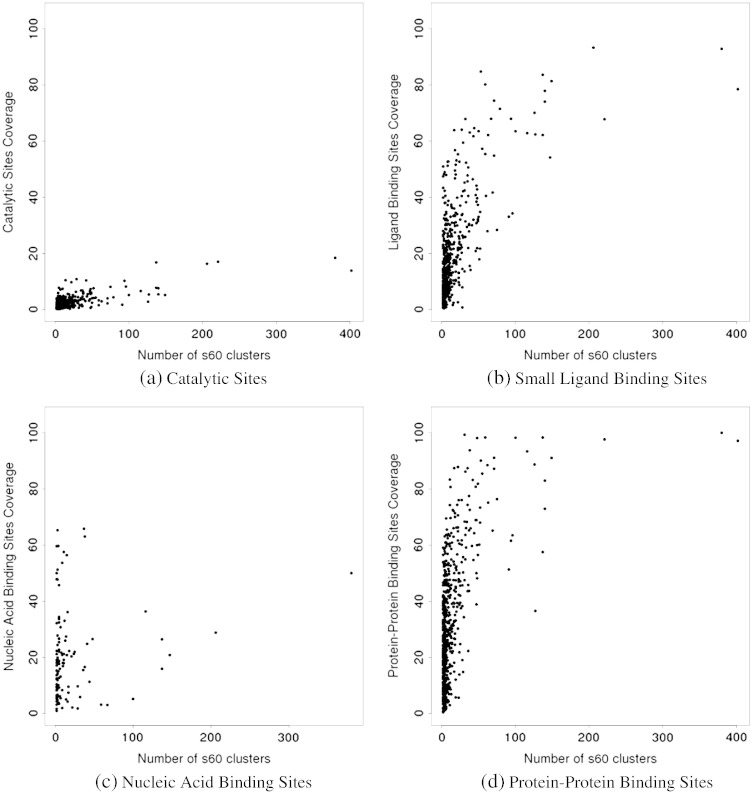
Functional site coverage and sequence diversity of domain superfamilies. Each plot shows the data for a specific type of functional site. Each superfamily is represented as a dot in these plots. Functional site coverage on the Y-axis is measured as the proportion of residues in the representative that map to at least one site in any member of the superfamily. Superfamily diversity on the X-axis is measured as the number of clusters of sequences at 60% sequence identity in the superfamily.

Fig. 2 shows the functional site coverage against a simple measure of superfamily diversity, namely the number of clusters of domains at 60% sequence identity. Four different types of functional sites, namely catalytic sites, binding sites for small ligands, nucleic acid-binding sites, and protein–protein interfaces, were considered separately.

We observe that coverage is rather limited for catalytic sites, confirming that these tend to co-occur always in the same general location within superfamilies. This can be explained in part by their small size, and from the fact that their location may be rather constrained by steric considerations, such as the need for a pocket in which the reaction is performed [32]. Supplementary material Fig. A.13 shows that the functional site coverage follows a highly similar trend when plotted against the number of domains in a superfamily.

In contrast, we observe large coverage of the representative by protein–protein binding sites, for a significant number of superfamilies. This indicates that in these superfamilies, protein–protein interfaces can occur in any topological location. Since we have summed protein–protein sites from all relatives in the superfamily onto the representative, this high coverage suggests that one or more relatives may have multiple partners with different sites and also that binding partners and their sites may differ between relatives. In particular, we can see that for the vast majority of superfamilies with significant diversity (e.g. more than 20 60% sequence identity clusters), more than 60% of residues in the representative map to protein–protein interfaces.

Binding sites for small ligands are more limited in their distribution, which would be expected from the fact that small ligands are generally smaller than macromolecules. Yet, we also note the presence of several superfamilies with large coverage. A handful of superfamilies have coverage values over 80%, and a significant fraction of superfamilies have coverage over 50%. This is somewhat unexpected as small-ligand binding sites are generally quite small by definition. However, it cannot be excluded that in some cases confounding factors may also play a role. For example, in some relatives in the P-loop superfamily, some artificial ligands have been used in a screening study that finds lots of potential binding sites.

Nucleic acid-binding site data is more sparse. Many superfamilies do not have any such binding sites, and only a few have coverage values above 30% for these types of sites. A large number of superfamilies have a representative coverage of 0 for nucleic acid binding, suggesting no domains within them are involved in that type of function. This illustrates the fact that nucleic-acid binding is a more unusual ability than binding other protein chains or small ligands.

Several DNA-binding domains exist within the promoter regions of transcription factors. These domains include zinc fingers, homeobox domains, helix-turn-helices and leucine zippers [47,48], which tend to favour particular sites. These sites are represented as conserved consensus sequence motifs [49].

### 3.2 Site coverage increases with sequence diversity in the superfamily

Generally, we note that superfamilies with significant diversity tend to display large functional site coverage values.

Supplementary material Fig. A.14 shows the functional site coverage firstly against the number of superfamilies with this coverage and secondly against the number of domain sequences in superfamilies with this coverage. Four different types of functional site have been analysed, as in Fig. 2.

The functional site coverage of catalytic residues in Supplementary material Fig. A.14a is limited, as previously shown in Fig. 2a. Across Supplementary material Fig. A.14, a trend can be observed where the superfamilies with very high functional site coverage are also the superfamilies with the highest number of domain sequences. In the small ligand binding sites (Supplementary material Fig. A.14b) for example, at 97% functional site coverage, there is a single superfamily, which has a total of 727,424 sequences, the largest number of domain sequences observed in this figure. At 99% functional site coverage in the protein–protein interface sites (Supplementary material Fig. A.14d), there are four superfamilies with a total of 129,941 domain sequences, which is a significant proportion of all the domain sequences. This suggests, as expected, that superfamilies with large numbers of sequences are more likely to have higher diversity of functional site location.

In the rest of the manuscript, we will mostly illustrate diversity by referring to the situation with protein–protein interfaces, and small ligand binding sites to a lesser extent.

### 3.3 Illustrations of functional site coverage

**Fig. 3:**
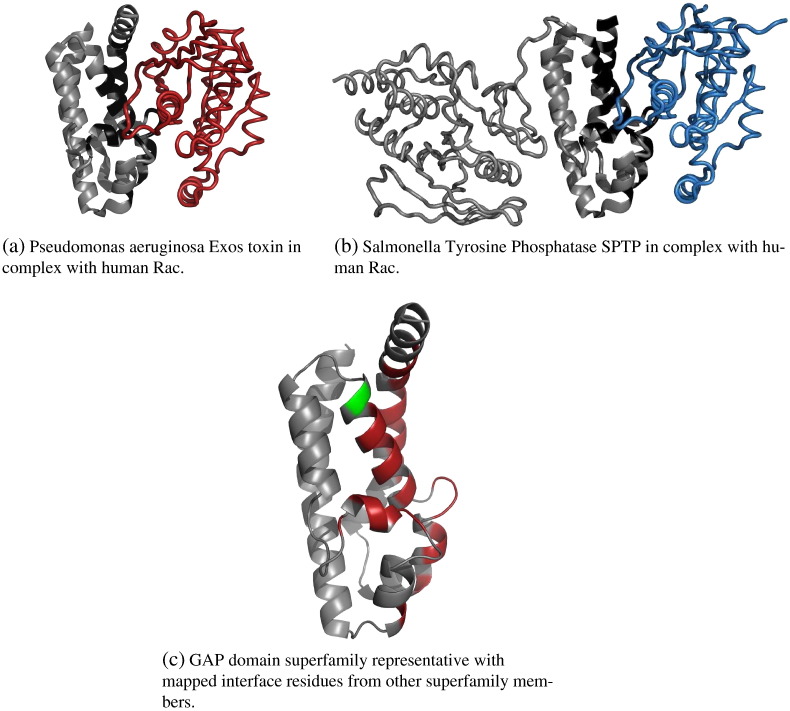
Example of a small superfamily with limited coverage of protein-protein interfaces. This is the Bacterial GTP-ase Activating Protein (GAP) domain superfamily (CATH code 1.20.120.260). The GAP domain is always displayed in grey cartoon. In Fig. 3a and 3b, the interacting partner is coloured red and blue, respectively. Interface residues on the GAP domain are coloured black. Fig. 3a and 3b display PDB entries 1he1 and 1g4u1he11g4u, respectively. In Fig. 3c, the representative is displayed in grey cartoons. Residues that map to interface residues in superfamily members are coloured according to the percentage of members that have an interface residue at that position, using the following colour scale: 0 in grey, 1–20% in blue, 20–40% in green, 40–60% in yellow, 60–80% in orange and 80–100% in red).

**Fig. 4:**
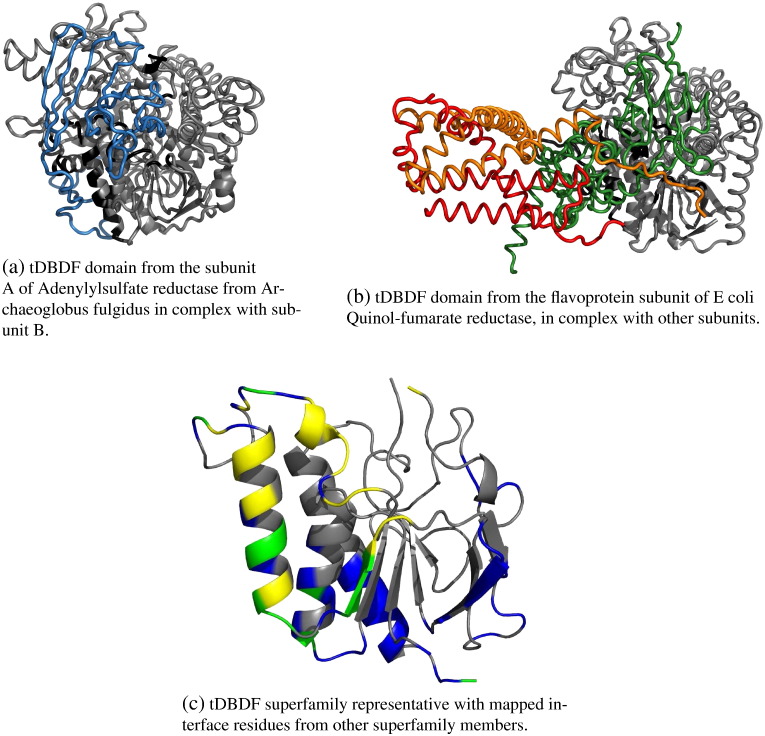
Example of a large and diverse superfamily with limited coverage of protein-protein interfaces. This is the “Two-Dinucleotide Binding Domains” Flavoprotein (tDBDF) superfamily (CATH code 3.50.50.60). The tDBDF domain is always displayed in grey cartoon. In Fig. 4a (PDB entry 1jnr) and 4b (PDB entry 1kf6), the interacting partners are represented as coloured traces. Interface residues on the tDBDF domain are coloured black. The interface occurs in a similar location in these two distinct domains. Fig. 4c shows the representative with residues coloured according to the fraction of superfamily members that have an interface residue at that position, following the same colour scheme as described at Fig. 3.

**Fig. 5:**
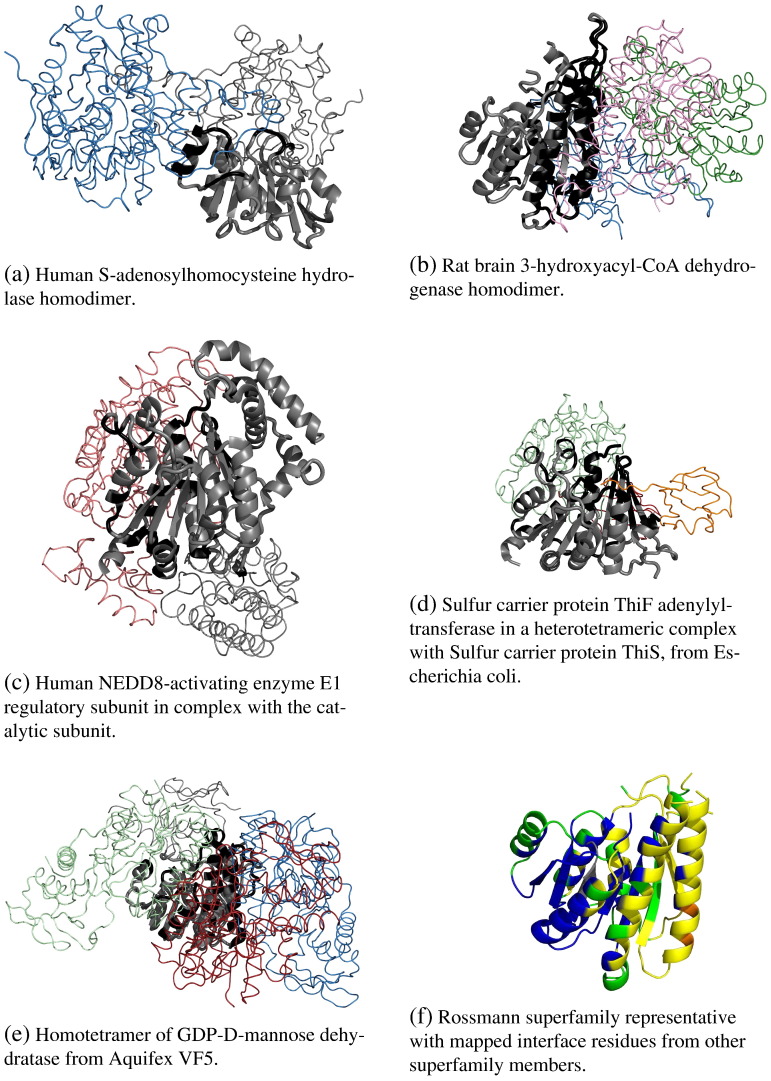
Example of a large and diverse superfamily with large coverage of protein-protein interfaces. This is the NAD(P)-binding Rossmann superfamily (CATH code.40.50.720). The Rossmann domain is always displayed in grey cartoon and shown in the same orientation. Extra-domains from the same chain are displayed as grey traces. Interacting partners are displayed as coloured traces. Interface residues on the Rossmann domain are coloured black. Fig. 5a through to 5e display PDB entries 1a7a, 1e3w, 1tt5, 1zud, and 2z1m, respectively. Fig. 5f shows the representative with residues coloured according to the same colour scheme as described at Fig. 3.

In order to help further understand Fig. 2, we illustrate them with examples taken from different regions of the coverage plots (see Fig. 3 to 5). We have chosen to focus on the specific case of protein–protein interfaces.

#### 3.3.1 Superfamilies with low site coverage and low sequence diversity

Fig. 3 illustrates the situation for the bacterial GAP domain superfamily, which is characterised by low diversity and low protein–protein interface coverage. This is a typical example of a superfamily that is not highly populated and not sequence diverse, and for which the interfaces in the different characterised members are found in similar locations.

The GTPase activating protein (GAP) is a type of bacterial effector injected into a eukaryotic host cell via the type III secretion system. Litvak and Selinger [50] examined a multiple sequence alignment of 10 bacterial GAPs and found two short highly conserved motifs across the different species, together with four completely conserved leucine residues. The two short motifs were found to provide an extensive network of interactions and structural constraints that are used to keep a catalytic arginine residue in its highly constrained and optimal position. These interactions include contacts with the p-loop of the GTPase, the GTPase switch regions, and with the bound nucleotide [50]. Fig. 3c shows a heat-map of the representative from the GAP domain superfamily with residue positions coloured according to the proportion of domains in the superfamily having a residue at that site involved in protein interactions. Both motifs are highlighted in red in Fig. 3c, showing that the residues within these motifs are involved in protein–protein interactions, and that their location is highly conserved throughout the superfamily.

Due to the high structural constraints imposed by the catalytic residues in this superfamily, and the nature of the interaction with the p-loop of the GTPases, it is not surprising to observe that the locations of protein–protein interactions across the superfamily members are highly similar in our analysis.

#### 3.3.2 Superfamily with low site coverage and high sequence diversity

Fig. 4 illustrates the protein–protein interface coverage for the “Two Dinucleotide Binding Domains” Flavoproteins (tDBDF) superfamily. This is a very large and diverse superfamily with 127 60% sequence identity clusters, but it has a limited coverage of protein–protein interfaces (37% of representative residues). This superfamily is exceptional in that most other large, diverse superfamilies have a protein–protein interface coverage of at least 50%. The bottom-right region of the plot is not occupied by many superfamilies (see Fig. 2d), suggesting as mentioned above, that with a few exceptions, diverse superfamilies have protein–protein interfaces covering most spatial locations in the domain structure.

Ojha et al. [51] performed a structural and functional analysis on 1664 members belonging to the tDBDF superfamily. As observed in our analysis, this enzymatic superfamily is very large and functionally diverse; members have previously been shown to catalyse many types of oxidation/reduction reactions in events such as energy metabolism, apoptosis, maintenance of redox homeostasis and cellular signalling. A wide variety of substrates are used to carry out these functions, which are either small molecules or proteins. All superfamily members have two dinucleotide binding Rossmann fold domains on a single chain, which both belong to the same CATH superfamily (CATH code 3.50.50.60). In order to function correctly, both domains are required; typically the N-terminal domain binds a flavin adenine dinucleotide (FAD) and the C-terminal domain binds a pyridine nucleotide. Despite a high level of functional diversity across the superfamily, (largely due to the variety of substrates), the position of these cofactors in the active site remains conserved so as to allow for optimal stereospecific hydride transfer between the two cofactors. The pyridine nucleotide is structurally restricted so that it has to interact with the FAD from the re-side, whose location is conserved across the superfamily. Due to the structural constraints placed upon the two cofactors, the geometry of the binding pockets is very highly conserved across the superfamily; the stacked configuration of the cofactors restricts the nicotinamide ring of the pyridine nucleotide from interacting with the isoalloxazine ring from the re-side of FAD [51]. This results in a single point of access to the FAD electron site, which in turn limits the number of residues that can be involved in interactions with acceptor proteins [51]. These constraints are reflected in our analysis where a limited number of residues are observed to be involved in protein–protein interactions.

#### 3.3.3 Superfamily with high site coverage and high sequence diversity

Fig. 5 illustrates the protein–protein interface coverage for the NAD(P)-binding Rossmann superfamily. This superfamily is extremely large with 402 60% sequence identity clusters. The NAD(P)-binding Rossmann domains bind the coenzyme nicotinamide adenine dinucleotide (NAD +) and a large selection of catalytic domains, which have been shown to come from at least seven different SCOP superfamilies [52]. NAD + is a co-factor in redox reactions in which the nicotinamide ring accepts a hydrogen in a reversible reaction. The type of catalytic domain that is bound to the Rossmann domain determines the substrate specificity and the exact catalytic reaction of the enzyme. Bashton and Chothia [52] discovered four different types of connections between catalytic and Rossmann domains depending on whether the catalytic domain occurred at the N- or C-terminus, in the middle of the Rossmann domain (Fig. 5c and e) or whether it includes the Rossmann domain within it (Fig. 5a). For the majority of the examples illustrated in the Bashton and Chothia paper [52], the catalytic domain lies close to the yellow region highlighted in Fig. 5f.

Fig. 5 shows a summary of the mapping of protein–protein interfaces on the superfamily representative. As this figure illustrates, protein–protein interfaces occur across a broad range of topological sites in this superfamily. Fig. 5a, for example represents the human S-adenosylhomocysteine (AdoHcy) hydrolase homodimer [53]. It consists of two chains, each comprising a NAD(P)-binding Rossmann domain nested within a catalytic domain. The method with which this enzyme binds to its cofactor, NAD, is thought to be unique and is important to its catalytic mechanism. Turner et al. [53] found that the dimer has a unique NAD-binding domain interface between helices α17 of the catalytic domain on one monomer and αC of the NAD(P)-binding Rossmann domain on the second monomer (black helix in Fig. 5a), and between α18 of the catalytic domain on one monomer and residues at the adenine side of the NAD binding site in the second monomer. In Fig. 5d the protein–protein interactions of ThiF differ from the previous example as it uses helices located towards the N-terminal part of ThiF to form a mostly hydrophobic interface with a second ThiF in the complex.

In spite of the fact that protein–protein interfaces occur at all topological sites in this superfamily, Fig. 5f shows that some regions (shown in yellow and orange) are clearly preferred over others (shown in blue). This phenomenon of preferential colocation shall be discussed further in the next section.

### 3.4 Functional site preferential colocation

The fact that coverage is high for a particular type of functional site (e.g. protein–protein interfaces), in a given superfamily, does not necessarily mean that all the locations are used equally often by different relatives, for function.

For example, as shown in Fig. 5, in relatives in the Rossmann domain superfamily discussed in Section 3.3.3 above, some protein partners bind close to the active site in the superfamily whilst others bind at more remote sites. Furthermore, different relatives are binding diverse partners at different remote sites.

Throughout our analysis, numbers of non-redundant domains that have a site mapping at any position on the representative are recorded, so that we can see if some positions are preferred over others.

Studying the tendency of functional sites to colocate in similar topological locations within superfamilies was not a direct aim of this analysis. Indeed, previous studies have reported the existence of preferred sites among remote homologues and even between structurally similar although not necessarily related proteins [6,7]. However, we used our dataset to perform a very simple analysis of preferential colocation.

**Fig. 6:**
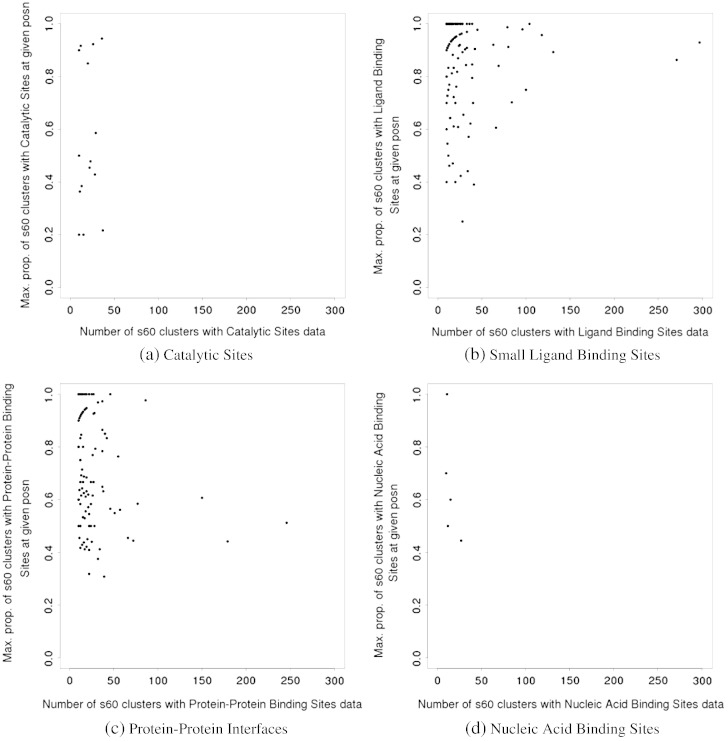
Preferential location of functional sites in CATH superfamilies. Each dot represents a superfamily. The plots show, on the Y-axis, the maximum proportion of 60% sequence identity clusters that have a functional site at a given position (or, in other words, it shows the proportion of 60% seq. id. clusters with a functional site at the position where that proportion is the highest). The X-axis shows the number of 60% seq. id. clusters that have functional site data of that type in the superfamily. Only superfamilies with at least 10 60% seq. id. clusters are considered here. This is to avoid meaningless fractions on the Y-axis (50% of 2 clusters is only one cluster).

The approach we followed was to verify whether there was, in each superfamily, at least one position where a majority of subfamilies (S60 clusters) have a functional site. As shown in Fig. 6 we observe that this seems to be the case for most superfamilies, thus confirming the existence of preferred locations.

The plots indicate that the majority of superfamilies have at least one position that is used as a functional site in at least 50% of the subfamilies (S60 clusters) in the superfamily. The constraints to produce these plots are rather strict as we considered only superfamilies with at least 10 60% sequence identity clusters with functional site data. This explains why the data is so sparse for nucleic-acid binding sites and catalytic sites. We chose these severe constraints to ensure that any preferential colocation of sites that was detected was not caused by under-sampling.

For catalytic sites and nucleic acid binding sites, the scarcity of data does not permit to draw any strong conclusion.

Whilst Fig. 6, suggesting preferential location, may appear to contradict the observation of high site coverage shown in Fig. 2, essentially there is no contradiction in the plots. A common binding site and high site coverage are both possible in a superfamily. This notably arises from the fact that a relative can bind multiple partners. So a common site may be used by many relatives but each of those relatives may bind additional partners at different sites.

As well as examining site preference in superfamilies, we also investigated whether the site coverage tends to be lower for a functional subfamily than for superfamilies i.e. lower site coverage for relatives sharing similar functional properties and therefore more likely to have similar protein partners. Supplementary material Fig. A.15 shows that most functional subfamilies examined have site coverage lower than 30% for protein interactions. The trends observed are generally much lower than observed for superfamilies (see Fig. 6 and Supplementary material Fig. A.16) supporting the idea that the higher site coverage observed when the whole superfamily is considered is largely due to the fact that functionally different relatives are exploiting different sites on the domain for binding ligand/protein partners.

**Fig. 7:**
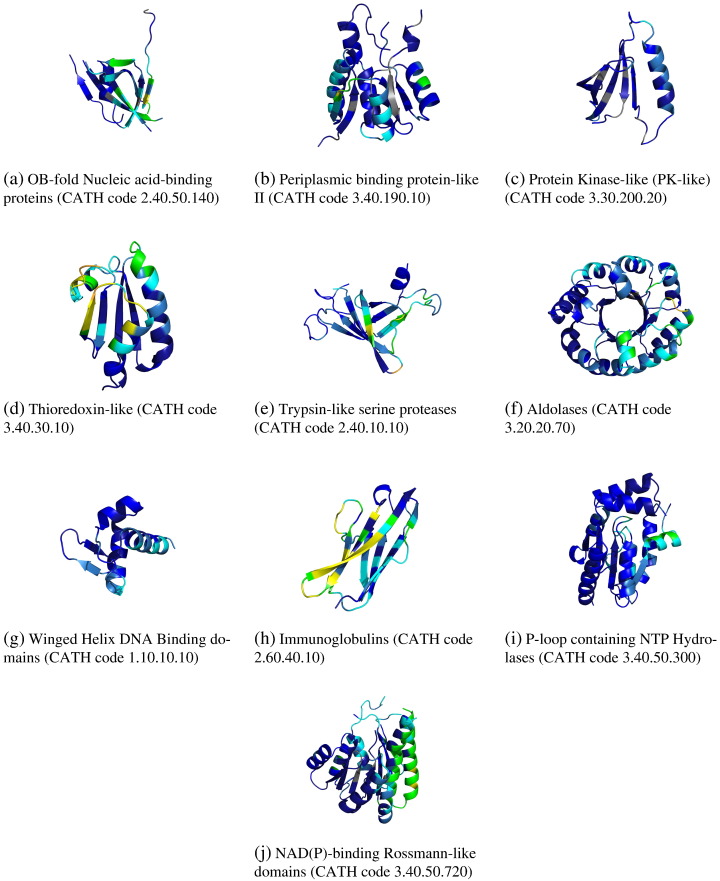
Protein-protein interface coverage for 10 most populated superfamilies in the CATH database. The colour scheme is the same as in Fig. 3.

In Fig. 7, we illustrate protein–protein interface coverage summaries for the most diverse superfamilies, to show what the different patterns of preferential colocation are. As can be seen, all of these superfamilies use most of their locations for protein–protein interfaces, but one region is generally preferred over others.

### 3.5 Functional site coverage compared with measures of function and structure diversity

Next, we attempt to interpret our results by using a number of measures of functional diversity and structural diversity of superfamilies and contrasting that with the coverage in functional sites.

For this, we check the distribution of superfamilies with different structural and functional features, and see if any signal is observed that may help identify superfamilies that have greater functional site location versatility.

#### 3.5.1 Site coverage of enzyme and non-enzyme superfamilies

First we tested whether enzymatic superfamilies differed from non-enzymatic superfamilies in terms of functional site coverage. The idea is that enzymes are generally thought to harbour one main canonical active site, therefore being perhaps less likely to have high coverage. However, there does not seem to be an obvious trend in the distribution of enzyme superfamilies in terms of functional site coverage of any type.

The lack of any obvious difference in coverage between enzyme and non-enzyme superfamilies may reflect the fact that whilst enzymes have a preferred active site for binding substrates, they also use other regions on the protein surface for binding protein partners e.g. in enzyme complexes. The plots are shown in Supplementary material Fig. A.17.

#### 3.5.2 Correlation of site coverage with tendency to be a hub protein

We then checked whether superfamilies that contain domains from protein interaction network hub proteins display different patterns of functional site plasticity. At least some categories of hubs are thought to interact with their many partners via several distinct sites on their surfaces. The distribution of hub-related superfamilies is shown in Supplementary material Fig. A.18. As can be seen from the plots, the hub superfamilies do not cluster in a particular area of the plot. Some hub superfamilies have low interface coverage, whereas others have very high coverage. This can be explained by a number of factors. First hub-related superfamilies may not always be directly involved in the interactions, because hubs are defined at the protein level, not the domain level. Second, structural data is missing for a large number of interactions so that although interactions are known, interfaces may not be. Finally, and as alluded to above, from a biological standpoint, hubs may be using the same interface for many of their interactions.

However, clearly information collected by our analyses may be helpful for other researchers investigating putative hub domains. For example, domains thought to be acting as hubs which belong to diverse superfamilies, with low site coverage, may exploit a single specific surface in their interactions which would allow them to regulate these interactions.

#### 3.5.3 Structural diversity

**Fig. 8:**
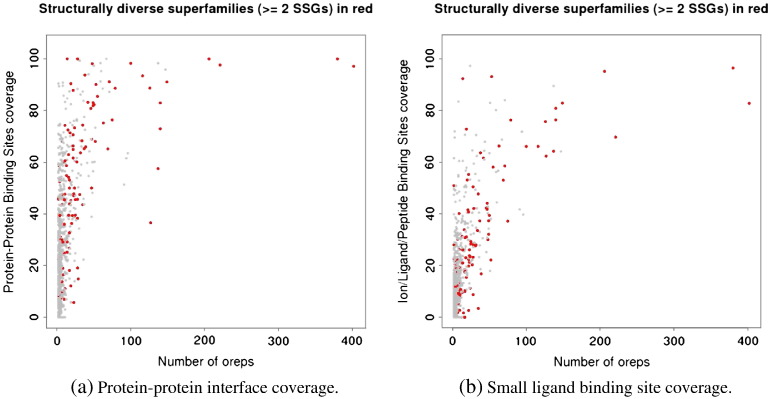
Functional site coverage versus superfamily diversity, with structurally diverse superfamilies coloured in red. Superfamilies are defined as structurally diverse if they contain at least 2 structural clusters (see Methods section).

When displaying the structural diversity of superfamilies on the plots shown in Fig. 2, we observe that structurally diverse superfamilies mostly have high site coverage (see Fig. 8). This is the case whether we measure structural diversity by the number of clusters generated using a 9.0 Å RMSD threshold or 5.0 Å RMSD threshold (see Supplementary material Fig. A.19 and A.20). Indeed, a Wilcoxon Rank-Sum test performed to compare the distributions of coverage values for structurally diverse superfamilies *vs* the other ones, shows that structurally diverse superfamilies have significantly higher coverage values, for all types of sites except nucleic acid binding sites (see Table 2).

Previous work has shown that one of the ways by which homologous domains were able to explore new regions of functional space, was by exploiting structural embellishments, for function. It was shown that these structural embellishments have a tendency to be directly involved in binding or catalysis [16,15]. It should however be noted that superfamilies with high site coverage are not necessarily the most diverse ones, suggesting that high site coverage is not exclusively a function of structural diversity.

Results here point at the existence of large and diverse superfamilies that may exploit different mechanisms for novel function exploration. Results also seem to show, at least for protein–protein interactions, that the very diverse superfamilies are very versatile in adapting different parts of their surface for interactions.

### 3.6 Conserved sites coverage

One problem with the known functional site data is that it is notoriously incomplete. Known functional sites are for the most part characterised by solving structures of proteins in complex with their partners. However, proteins of known structures only represent a very small fraction of known proteins. Furthermore, even for proteins for which a structure is available, many binding sites may still be unknown.

In order to account for that problem, we have performed a similar analysis as described above, but this time using a set of predicted functional residues, i.e. residues that are conserved in functional families of protein domains. We map these conserved residues onto superfamily representatives in the same way as for known functional residues.

For this part, we first needed to confirm that the conserved residues identified in our functional families of protein domains are indeed appropriate proxies for functional residues. To do this, we evaluated the extent to which such conserved residues overlapped with the known functional sites whenever these were available. For all functional families where known functional residues were available, we calculated the proportion of such residues being recovered by our conserved residues.

**Fig. 9:**
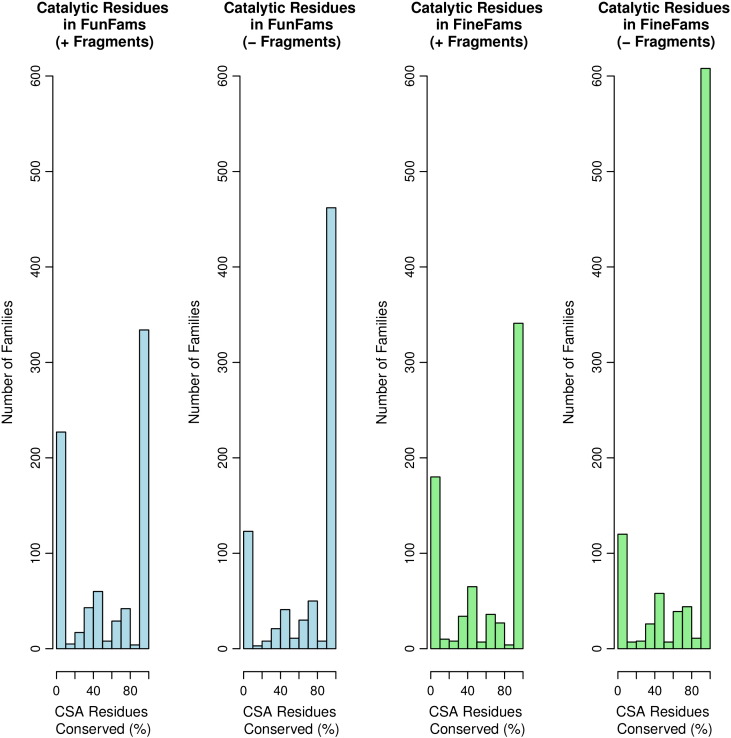
Comparison of the number of catalytic residues that are conserved in each type of functional family, before and after removing fragments.

For catalytic residues, we found that about 58% and 60% were conserved in FunFam and FineFam alignments, respectively. Following the removal of sequence fragments from these alignments, this value rose to about 76% and 78% for FunFams and FineFams, respectively. Comparing the two types of functional family, more conserved catalytic residues are observed in the FineFams (Fig. 9).

For binding residues, we found that protein–protein interface residues are the least often conserved across functional family types, whereas the small ligand binding residues are the most often conserved (Table 3). We also found that the number of conserved functional residues increased in both family types following the removal of fragments. Following sequence fragment removal, the FineFams showed the highest levels of functional residue conservation, showing that conserved residues in this type of functional family are better proxies for functional residues.

We also performed enrichment tests as described in the Methods section, to ensure that conserved residues are enriched in functional residues when compared to the background set of residues. Wilcoxon Rank-Sum tests performed over the enrichment values provided highly significant P-values for all functional site types except IBIS protein–protein interface residues, suggesting that conserved residues are highly enriched in the remaining three types of functional residues when compared to all residues: catalytic residues, nucleic acid binding residues, and small ligand binding residues (see Table 4).

When we use these conserved residues to perform our analysis of site coverage within superfamilies (as presented in Fig. 2 for known sites), we observe a distribution of coverage values that confirms the existence of an important diversity of site locations in many superfamilies (see Supplementary material Fig. A.21, and Table 5).

## 4 Discussion

Our results show that although there is sometimes a preference for at least one residue site in a superfamily, other sites can be exploited usually by protein partners and particularly in diverse superfamilies a large proportion of the surface has been sampled in this way, by different relatives.

If we partition the superfamily into more functionally coherent subfamilies, we see that there is a greater tendency to exploit a common site (see Supplementary material Fig. A.15 and A.16). Therefore, the increase in coverage obtained when accumulating site data from all relatives across a superfamily, is due to the fact that some of the functional subfamilies must exploit different sites on the domain surface.

These observations (i.e. preferred sites and multiple sites across the domain) are not contradictory. A common binding site and high surface coverage are both possible in a superfamily. This notably arises from the fact that a relative can bind multiple partners. So a common site may be used by many relatives but each of those relatives may bind additional partners at different sites.

For example, in an enzyme superfamily, a domain may bind multiple protein partners in an enzyme complex or oligomeric unit. One of these partners may bind at the common superfamily active site as it may contribute additional catalytic residues to the site. Another may bind at a different site, quite remote from the common active site. Summed over the whole superfamily this can result in one or more common sites used by many relatives but also many additional unique sites over the surface corresponding to the binding sites of different ligands/partners bound by different relatives.

Therefore, for the protein–protein interaction plots, the large coverage i.e. diversity of sites being exploited by different relatives, could be associated with changes in the specificity of the proteins that they bind. Previous analyses on protein families in yeast and *Escherichia coli*, have shown by analysing GO terms, that different paralogous relatives within a superfamily tend to bind to different protein partners [54]. Our results here may suggest that these diverse protein partners bind at different sites on the domain surface. Similarly, when considering an individual relative with multiple protein partners, it may be beneficial to have these partners binding at different sites, to reduce cross effects, unless binding to a common site was somehow important for regulation.

We believe that our observations relate to both physical considerations and evolutionary ones. For example, in enzymes, one of the deepest surface pockets are frequently exploited for binding substrates [55] and this is often used by all the relatives. This site is therefore highly conserved throughout evolution, presumably because it has the best features to support a chemical reaction.

However, for metal binding or protein interactions where the activity does not need to be so exquisitely tailored to provide the precise stereochemistry of catalytic residues, most parts of the surface may be able to evolve the right characteristics for binding ligands/protein partners. Therefore different functional surfaces can emerge during evolution in duplicated domains.

Relatives in different organisms often bind different metal ions and can sometimes exploit different co-factors. The fact that it appears possible for domains to change their surfaces during evolution to bind different molecules clearly contributes to the diversity in functional repertoires that we see between different species.

Our analyses have been purely empirical and reflect what is currently observed in the PDB when all relatives with known site data are sampled. They are not intended to infer probabilities of binding to particular sites.

There are a number of issues to keep in mind when interpreting our results. First, the diversity of site locations we present in this work is likely to be a lower bound. There are relatively few known functional sites in biological databases. For example, if you consider all the known site data available for the functional subfamilies in our analysis, Supplementary material Fig. A.22 shows that in the majority of superfamilies only 30% or less of the subfamilies have annotations. The reason for this lack of site data is that the main experimental method for identifying functional residues is still X-ray crystallography with a bound ligand. These data can be difficult to obtain, and therefore, for many proteins, functional residues are yet unknown.

For example, there could be transient complexes formed between a domain relative and protein partners. Our current observation on the proportions of relatives exploiting a common site will generally not take account of these protein partners because of the difficulty of capturing transient interactions. If these transient partners all exploit the same site, the tendency to use a preferred site within a superfamily becomes higher.

Secondly, our analysis relies on structural alignment between domains for functional residue mapping. We believe that by using the approach described here, we have minimized the risks of mis-alignments between residues of the representative and those of other domains in the superfamily. Indeed, we rely on structural alignments which are generally safer than sequence alignments, we use pairwise alignments rather than the more difficult multiple structure alignments, and we focus on comparisons between individual domains within superfamilies. This should generally ensure alignments that are as reliable-as-it-gets. However, we cannot rule out odd mis-mappings between residues, especially when comparing very different domains. In general, however, we expect such mis-mappings to occur between relatively small windows around the residues of interest.

Third, IBIS uses a distance cut-off of 4 Å from the ligand to define binding residues. This cut-off may sometimes slightly over-estimate the amount of residues to include in functional sites. However, similar and sometimes larger distance cut-offs are routinely used for defining functional sites. We trust that in the majority of cases, this is a reasonable threshold to be used.

Fourth, structural diversity within superfamilies means that some members may sometimes include large insertions or embellishments that are not present in other members. In particular, the representative may, more often than not, not include some of the embellishments that are present in other members of the superfamily. In those cases, we are potentially missing all the functional sites that occur on these embellishments, when we map functional residues on the representative. Again, this results in under-estimating functional site diversity across the superfamily. Given the way we chose the representative, we should minimize the risk of having the opposite situation, i.e. cases where the representative contains embellishments that are not present in other members of the superfamily. Indeed, the presence of such embellishments reduce overall structural similarity to other superfamily members, thus reducing the chance of a domain with large embellishments being selected as representative. As a reminder, the representative is chosen as the domain with the largest cumulative structural similarity to all other members of the superfamily. In addition, even if a representative with large and unique embellishments is selected, this means that once again we underestimate the real diversity of functional sites, since that would mean that those embellishments cannot map to functional sites in other domains, and therefore, the representative coverage in functional sites diminishes.

## 5 Conclusion

In this work, we have performed a comprehensive analysis of all superfamilies for which there is sufficient data, to check whether functional sites are constrained to a few locations or can appear anywhere on the domain.

We provide quantitative analyses that document superfamilies in which related domains differ in terms of the location of their functional sites. We show that this phenomenon is pervasive and actually occurs in a significant number of superfamilies, especially those that contain the largest numbers of domains. In particular, we demonstrate that if a superfamily reaches a certain level of functional diversity, functional site location diversity also ensues.

This diversity in functional sites has consequences in the way we understand proteins, in particular their functional plasticity. It also has consequences on the approaches used for predicting functional sites. Since it appears that functional sites can sometimes occur in many different locations among related domains, relying too strictly on homology transfer for functional site prediction may sometimes result in missing potentially interesting predictions. In that context, prediction methods that rely not only on transfer by homology but also use a range of other features such as structural and physico-chemical properties of residues, may also be helpful.

Our data can also be useful in highlighting which superfamilies are particularly versatile in terms of their functional site location. All our data on functional site diversity within superfamilies are available from the CATH website.

## Conflict of interest statement

None declared.

## Figures and Tables

**Table 1:** Number of superfamilies considered in the dataset for each type of functional site, after applying all filters.

Site type	#Superfamilies
Catalytic sites	328
Protein–protein interfaces	645
Nucleic acid binding sites	116
Small ligand binding sites	659

**Table 2:** Results from a Wilcoxon Rank-Sum test comparing functional site coverage values for structurally diverse superfamilies versus structurally similar superfamilies.

Site type	P-value
Catalytic sites	4.894147 × 10^− 19^
Protein–protein interfaces	9.74794 × 10^− 18^
Nucleic acid binding sites	0.04609505
Small ligand binding sites	6.176701 10^− 16^

**Table 3:** The proportion of IBIS functional residues that are also conserved.

Family type	IBIS nucleic acid binding	IBIS protein–protein interface	IBIS small ligand binding
FunFams (+ fragments)	26.15%	21.50%	31.63%
FunFams (− fragments)	37.32%	33.76%	47.41%
FineFams (+ fragments)	34.70%	24.34%	35.02%
FineFams (− fragments)	47.73%	39.28%	52.03%

**Table 4:** This table shows the P-values calculated from enrichment scores using Wilcoxon Rank-Sum tests. Catalytic, IBIS nucleic acid binding, and IBIS small ligand binding residue P-values are consistently highly significant, suggesting that the conserved residues in both family types are highly enriched in these three types of functional residues.

Family type	Catalytic residues	IBIS nucleic acid binding	IBIS protein–protein interface	IBIS small ligand binding
FunFams (+ fragments)	< 2.2 × 10^− 16^	5.874 × 10^− 06^	0.5516	< 2.2 × 10^− 16^
FunFams (− fragments)	< 2.2 × 10^− 16^	3.555 × 10^− 07^	0.3546	< 2.2 × 10^− 16^
FineFams (+ fragments)	< 2.2 × 10^− 16^	7.047 × 10^− 09^	0.009475	< 2.2 × 10^− 16^
FineFams (− fragments)	< 2.2 × 10^− 16^	2.187 × 10^− 06^	0.0006779	< 2.2 × 10^− 16^

**Table 5:** Proportion of superfamilies where the site coverage on the representative is higher than 50%. The numbers between brackets are the actual fractions.

Site type	Proportion of high coverage superfamilies
Catalytic sites	0% (0/328)
Protein–protein interfaces	20.5% (132/645)
Nucleic acid binding sites	7.8% (9/116)
Small ligand binding sites	8.0% (53/659)
Conserved sites (FineFams)	38.8% (541/1393)
Conserved sites (FunFams)	22.4% (299/1333)

## Appendix A Supplementary data

Supplementary material describes functional site diversity in protein domain superfamilies.

Figure A.10 Mean RMSD in structurally diverse superfamilies.

Figure A.11 Example of a conserved structural core between remote homologues.

Figure A.12 Functional site coverage versus number of domains of known structure.

Figure A.13 Functional site coverage versus number of domains of known structure (filtered dataset).

Figure A.14 Functional site coverage versus number of domain sequences.

Figure A.15 Functional site coverage in FineFam functional families of the HUP superfamily.

Figure A.16 Median functional site coverage for FineFam functional families within a superfamily.

Figure A.17 Functional site coverage for enzyme and non-enzyme superfamilies.

Figure A.18 Functional site coverage for hub-related superfamilies.

Figure A.19 Functional site coverage for structurally diverse superfamilies (SSG5).

Figure A.20 Distributions of functional site coverage between structurally diverse and structurally conserved superfamilies (SSG5).

Figure A.21 Conserved site coverage.

Figure A.22 Proportion of functional families with experimentally characterised functional sites.

## References

[1] Ezkurdia L. Bartoli, Fariselli P., Casadio R., Valencia A., Tress M.L. (2009). Progress and challenges in predicting protein–protein interaction sites. Brief. Bioinform..

[2] Aloy P., Ceulemans H., Stark A., Russell R.B. (2003). The relationship between sequence and interaction divergence in proteins. J. Mol. Biol..

[3] López G., Valencia A., Tress M.L. (2007). firestar—prediction of functionally important residues using structural templates and alignment reliability. Nucleic Acids Res..

[4] Roy A., Kucukural A., Zhang Y. (2010). I-TASSER: a unified platform for automated protein structure and function prediction. Nat. Protoc..

[5] Roy A., Zhang Y. (2012). Recognizing protein-ligand binding sites by global structural alignment and local geometry refinement. Structure.

[6] Russell R.B., Sasieni P.D., Sternberg M.J. (1998). Supersites within superfolds. Binding site similarity in the absence of homology. J. Mol. Biol..

[7] Zhang Q.C., Petrey D., Norel R., Honig B.H. (2010). Protein interface conservation across structure space. Proc. Natl. Acad. Sci..

[8] Chothia C., Gough J. (2009). Genomic and structural aspects of protein evolution. Biochem. J..

[9] Cuff A.L., Sillitoe I., Lewis T., Clegg A.B., Rentzsch R., Furnham N., Pellegrini-Calace M., Jones D., Thornton J., Orengo C.A. (2011). Extending CATH: increasing coverage of the protein structure universe and linking structure with function. Nucleic Acids Res..

[10] Andreeva A., Howorth D., Chandonia J.-M.M., Brenner S.E., Hubbard T.J., Chothia C., Murzin A.G. (2008). Data growth and its impact on the SCOP database: new developments. Nucleic Acids Res..

[11] Wass M.N., David A., Sternberg M.J. (2011). Challenges for the prediction of macromolecular interactions. Curr. Opin. Struct. Biol..

[12] Todd A.E., Orengo C.A., Thornton J.M. (2001). Evolution of function in protein superfamilies, from a structural perspective. J. Mol. Biol..

[13] Goldstein R.A. (2008). The structure of protein evolution and the evolution of protein structure. Curr. Opin. Struct. Biol..

[14] Redfern O., Dessailly B., Orengo C. (2008). Exploring the structure and function paradigm. Curr. Opin. Struct. Biol..

[15] Reeves G.A., Dallman T.J., Redfern O.C., Akpor A., Orengo C.A. (2006). Structural diversity of domain superfamilies in the CATH database. J. Mol. Biol..

[16] Dessailly B.H., Redfern O.C., Cuff A.L., Orengo C.A. (2010). Detailed analysis of function divergence in a large and diverse domain superfamily: toward a refined protocol of function classification. Structure.

[17] Korkin D., Davis F.P., Sali A. (2005). Localization of protein-binding sites within families of proteins. Protein Sci..

[18] Addou S., Rentzsch R., Lee D., Orengo C.A. (2009). Domain-based and family-specific sequence identity thresholds increase the levels of reliable protein function transfer. J. Mol. Biol..

[19] Lees J., Yeats C., Perkins J., Sillitoe I., Rentzsch R., Dessailly B.H., Orengo C. (2012). Gene3D: a domain-based resource for comparative genomics, functional annotation and protein network analysis. Nucleic Acids Res..

[20] Lee D.A., Rentzsch R., Orengo C. (2010). GeMMA: functional subfamily classification within superfamilies of predicted protein structural domains. Nucleic Acids Res..

[21] Rentzsch R., Orengo C. (2013). Protein function prediction using domain families. BMC Bioinforma.

[22] Li W., Godzik A. (2006). Cd-hit: a fast program for clustering and comparing large sets of protein or nucleotide sequences. Bioinformatics.

[23] Katoh K., Kuma K.-i., Toh H., Miyata T. (2005). MAFFT version 5: improvement in accuracy of multiple sequence alignment. Nucleic Acids Res..

[24] Sadreyev R., Grishin N. (2003). COMPASS: a tool for comparison of multiple protein alignments with assessment of statistical significance. J. Mol. Biol..

[25] Pegg S.C.-H.C., Brown S.D., Ojha S., Seffernick J., Meng E.C., Morris J.H., Chang P.J., Huang C.C., Ferrin T.E., Babbitt P.C. (2006). Leveraging enzyme structure–function relationships for functional inference and experimental design: the structure–function linkage database. Biochemistry.

[26] Porter C.T., Bartlett G.J., Thornton J.M. (2004). The catalytic site atlas: a resource of catalytic sites and residues identified in enzymes using structural data. Nucleic Acids Res..

[27] Shoemaker B.A., Zhang D., Tyagi M., Thangudu R.R., Fong J.H., Marchler-Bauer A., Bryant S.H., Madej T., Panchenko A.R. (2012). IBIS (inferred biomolecular interaction server) reports, predicts and integrates multiple types of conserved interactions for proteins. Nucleic Acids Res..

[28] Krissinel E., Henrick K. (2007). Inference of macromolecular assemblies from crystalline state. J. Mol. Biol..

[29] Berman H., Henrick K., Nakamura H. (2003). Announcing the worldwide protein data bank. Nat. Struct. Mol. Biol..

[30] Orengo C.A., Taylor W.R. (1996). [36] SSAP: sequential structure alignment program for protein structure comparison. Vol. 266 of Methods in Enzymology.

[31] Redfern O.C., Dessailly B.H., Dallman T.J., Sillitoe I., Orengo C.A. (2009). FLORA: a novel method to predict protein function from structure in diverse superfamilies. PLoS Comput. Biol..

[32] Bartlett G.J., Porter C.T., Borkakoti N., Thornton J.M. (2002). Analysis of catalytic residues in enzyme active sites. J. Mol. Biol..

[33] Katoh K., Toh H. (2008). Recent developments in the MAFFT multiple sequence alignment program. Brief. Bioinform..

[34] Valdar W.S.J. (2002). Scoring residue conservation. Proteins.

[35] Rausell A., Juan D., Pazos F., Valencia A. (2010). Protein interactions and ligand binding: from protein subfamilies to functional specificity. Proc. Natl. Acad. Sci..

[36] Kruskal W.H. (1957). Historical notes on the Wilcoxon unpaired two-sample test. J. Am. Stat. Assoc..

[37] R Core Team (2012). R: a language and environment for statistical computing. http://www.R-project.org.

[38] Kerrien S., Aranda B., Breuza L., Bridge A., Broackes-Carter F., Chen C., Duesbury M., Dumousseau M., Feuermann M., Hinz U., Jandrasits C., Jimenez R.C., Khadake J., Mahadevan U., Masson P., Pedruzzi I., Pfeiffenberger E., Porras P., Raghunath A., Roechert B., Orchard S., Hermjakob H. (2012). The IntAct molecular interaction database in 2012. Nucleic Acids Res..

[39] Licata L., Briganti L., Peluso D., Perfetto L., Iannuccelli M., Galeota E., Sacco F., Palma A., Nardozza A.P.P., Santonico E., Castagnoli L., Cesareni G. (2012). MINT, the molecular interaction database: 2012 update. Nucleic Acids Res..

[40] Stark C., Breitkreutz B.-J.J., Chatr-Aryamontri A., Boucher L., Oughtred R., Livstone M.S., Nixon J., Van Auken K., Wang X., Shi X., Reguly T., Rust J.M., Winter A., Dolinski K., Tyers M. (2011). The BioGRID interaction database: 2011 update. Nucleic Acids Res..

[41] Xenarios I., Salwínski L., Duan X.J.J., Higney P., Kim S.-M.M., Eisenberg D. (2002). DIP, the database of interacting proteins: a research tool for studying cellular networks of protein interactions. Nucleic Acids Res..

[42] Goel R., Harsha H.C., Pandey A., Prasad K.S. (2012). Human protein reference database and human proteinpedia as resources for phosphoproteome analysis. Mol. Biosyst..

[43] Croft D., O'Kelly G., Wu G., Haw R., Gillespie M., Matthews L., Caudy M., Garapati P., Gopinath G., Jassal B., Jupe S., Kalatskaya I., Mahajan S., May B., Ndegwa N., Schmidt E., Shamovsky V., Yung C., Birney E., Hermjakob H., D'Eustachio P., Stein L. (2011). Reactome: a database of reactions, pathways and biological processes. Nucleic Acids Res..

[44] Navratil V., de Chassey B., Meyniel L., Delmotte S., Gautier C., André P., Lotteau V., Rabourdin-Combe C. (2009). VirHostNet: a knowledge base for the management and the analysis of proteome-wide virusâ€“host interaction networks. Nucleic Acids Res..

[45] Flicek P., Amode M.R., Barrell D., Beal K., Brent S., Carvalho-Silva D., Clapham P., Coates G., Fairley S., Fitzgerald S., Gil L., Gordon L., Hendrix M., Hourlier T., Johnson N., Kähäri A.K., Keefe D., Keenan S., Kinsella R., Komorowska M., Koscielny G., Kulesha E., Larsson P., Longden I., McLaren W., Muffato M., Overduin B., Pignatelli M., Pritchard B., Riat H.S., Ritchie G.R.S., Ruffier M., Schuster M., Sobral D., Tang Y.A., Taylor K., Trevanion S., Vandrovcova J., White S., Wilson M., Wilder S.P., Aken B.L., Birney E., Cunningham F., Dunham I., Durbin R., Fernández-Suarez X.M., Harrow J., Herrero J., Hubbard T.J.P., Parker A., Proctor G., Spudich G., Vogel J., Yates A., Zadissa A., Searle S.M.J. (2012). Ensembl 2012. Nucleic Acids Res..

[46] Dessailly B.H., Nair R., Jaroszewski L., Fajardo J.E., Kouranov A., Lee D., Fiser A., Godzik A., Rost B., Orengo C. (2009). PSI-2: structural genomics to cover protein domain family space. Structure.

[47] Cho S.Y., Chung M., Park M., Park S., Lee Y.S. (2008). ZIFIBI: prediction of DNA binding sites for zinc finger proteins. Biochem. Biophys. Res. Commun..

[48] Wilson D., Charoensawan V., Kummerfeld S.K., Teichmann S.A. (2008). DBDâ€“â€”taxonomically broad transcription factor predictions: new content and functionality. Nucleic Acids Res..

[49] Stormo G.D. (2000). DNA binding sites: representation and discovery. Bioinformatics.

[50] Litvak Y., Selinger Z. (2003). Bacterial mimics of eukaryotic GTPase-activating proteins (GAPs). Trends Biochem. Sci..

[51] Ojha S., Meng E.C., Babbitt P.C. (2007). Evolution of function in the “two dinucleotide binding domains” flavoproteins. PLoS Comput. Biol..

[52] Bashton M., Chothia C. (2002). The geometry of domain combination in proteins. J. Mol. Biol..

[53] Turner M.A., Yuan C.-S., Borchardt R.T., Hershfield M.S., Smith G.D., Howell P.L. (1998). Structure determination of selenomethionyl S-adenosylhomocysteine hydrolase using data at a single wavelength. Nat. Struct. Mol. Biol..

[54] Reid A., Ranea J., Orengo C. (2010). Comparative evolutionary analysis of protein complexes in E. coli and yeast. BMC Genomics.

[55] Laskowski R.A., Luscombe N.M., Swindells M.B., Thornton J.M. (1996). Protein clefts in molecular recognition and function. Protein Sci..

