# Supplementary material for: Functional site plasticity in domain superfamilies
Source: Biochim Biophys Acta. 2013 May;1834(5):874–89. doi: 10.1016/j.bbapap.2013.02.042 (PMC3787744; doi:10.1016/j.bbapap.2013.02.042)
Supplement: Figure A.10 — Mean RMSD in structurally diverse superfamilies. [file mmc1.pdf]

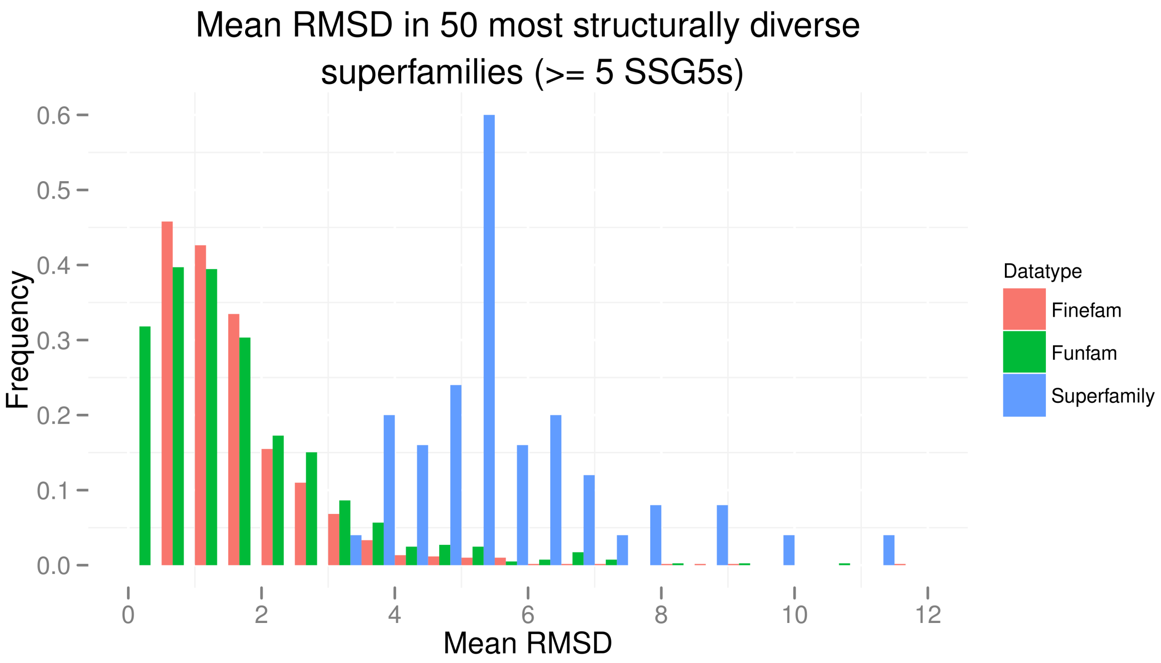

Figure A.10: The mean RMSD distributions calculated for FunFams, FineFams, and superfamilies within the top 50 most structurally diverse CATH superfamilies. Structural diversity is measured here by a superfamily have five or more structural clusters, or Structurally Similar Groups (SSGs), generated at 5Å. Using a Wilcoxon Rank-Sum test, the FunFam and FineFam distributions were found to be significantly different with a p-value of 0.0002253.

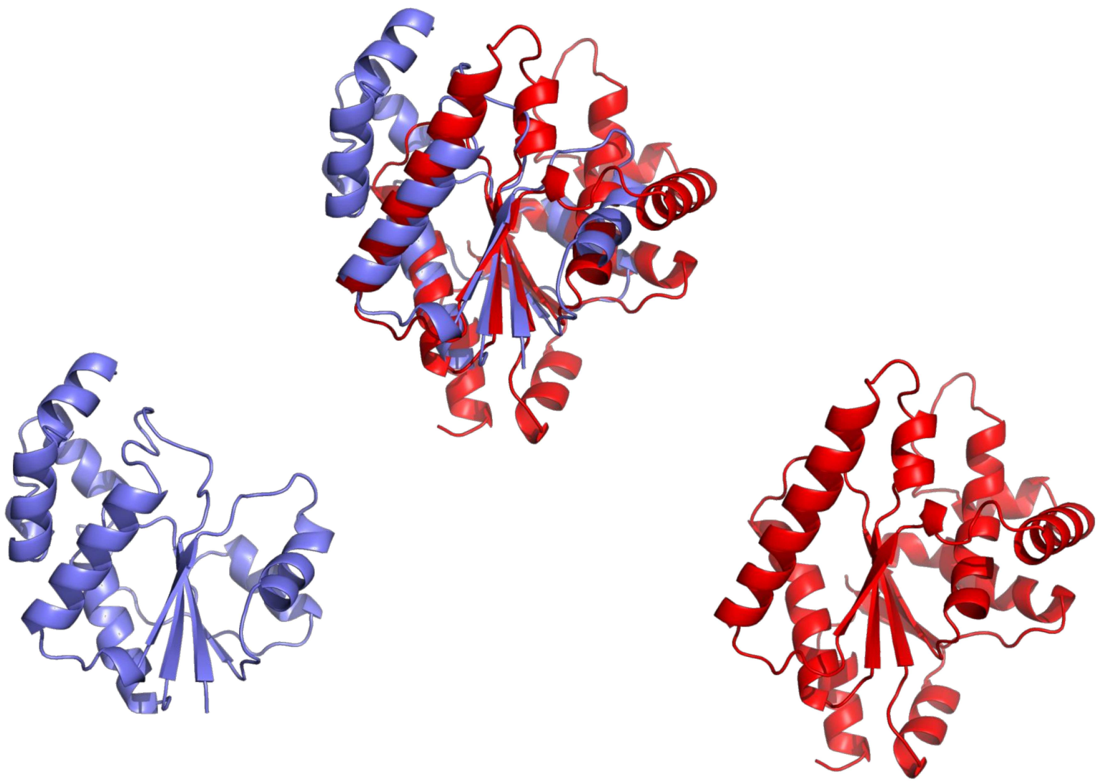

Figure A.11: Two very diverse relatives (sequence identity 6%, RMSD 14Å) from the highly structurally diverse HUPs superfamily (3.40.50.620). The conserved core can be clearly seen upon superposing the two domains, 1ej2A00 (light blue) and 1n3lA01 (red).

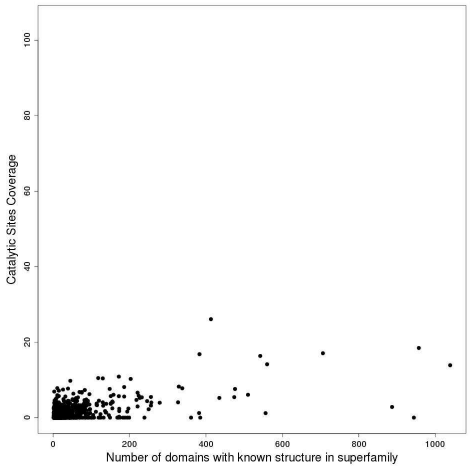

(a) Catalytic site coverage.

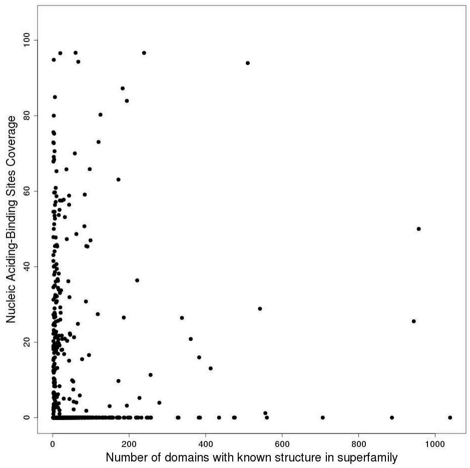

(b) Nucleic acid binding site coverage.

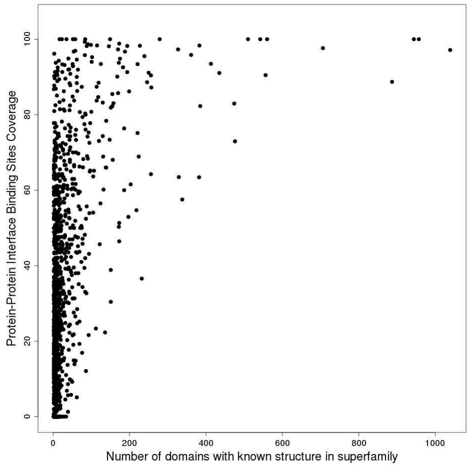

(c) Protein-protein binding site coverage.

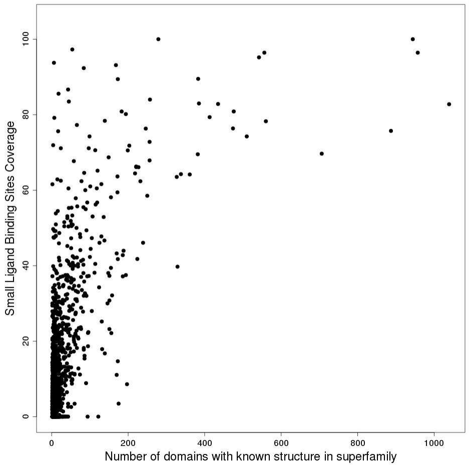

(d) Small ligand binding site coverage.

Figure A.12: Functional site coverage and domain count for superfamilies. Each plot shows the data for a specific type of functional site. Each superfamily is represented as a dot. These data show the functional site coverage before the filters were applied; superfamilies with a representative less than 100 amino acids were removed and also superfamilies with one domain contributing to more than 50% of the functional site coverage.

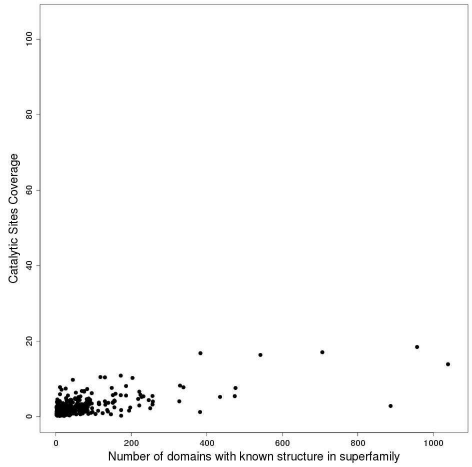

(a) Catalytic site coverage.

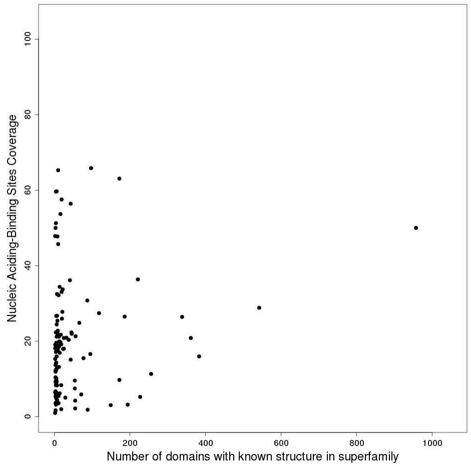

(b) Nucleic acid binding site coverage.

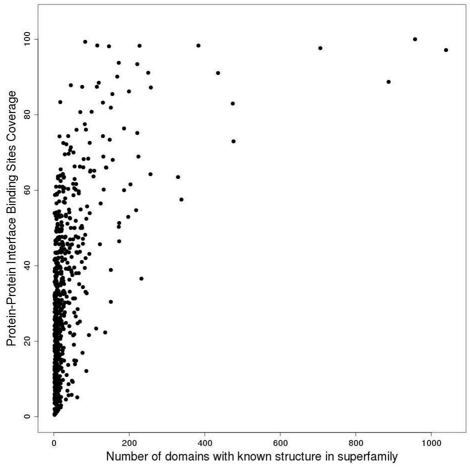

(c) Protein-protein binding site coverage.

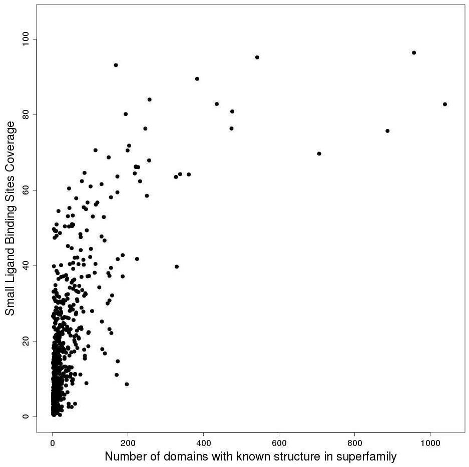

(d) Small ligand binding site coverage.

Figure A.13: Functional site coverage and domain count for superfamilies. Each plot shows the data for a specific type of functional site. Each superfamily is represented as a dot. These data show the functional site coverage after the filters were applied.

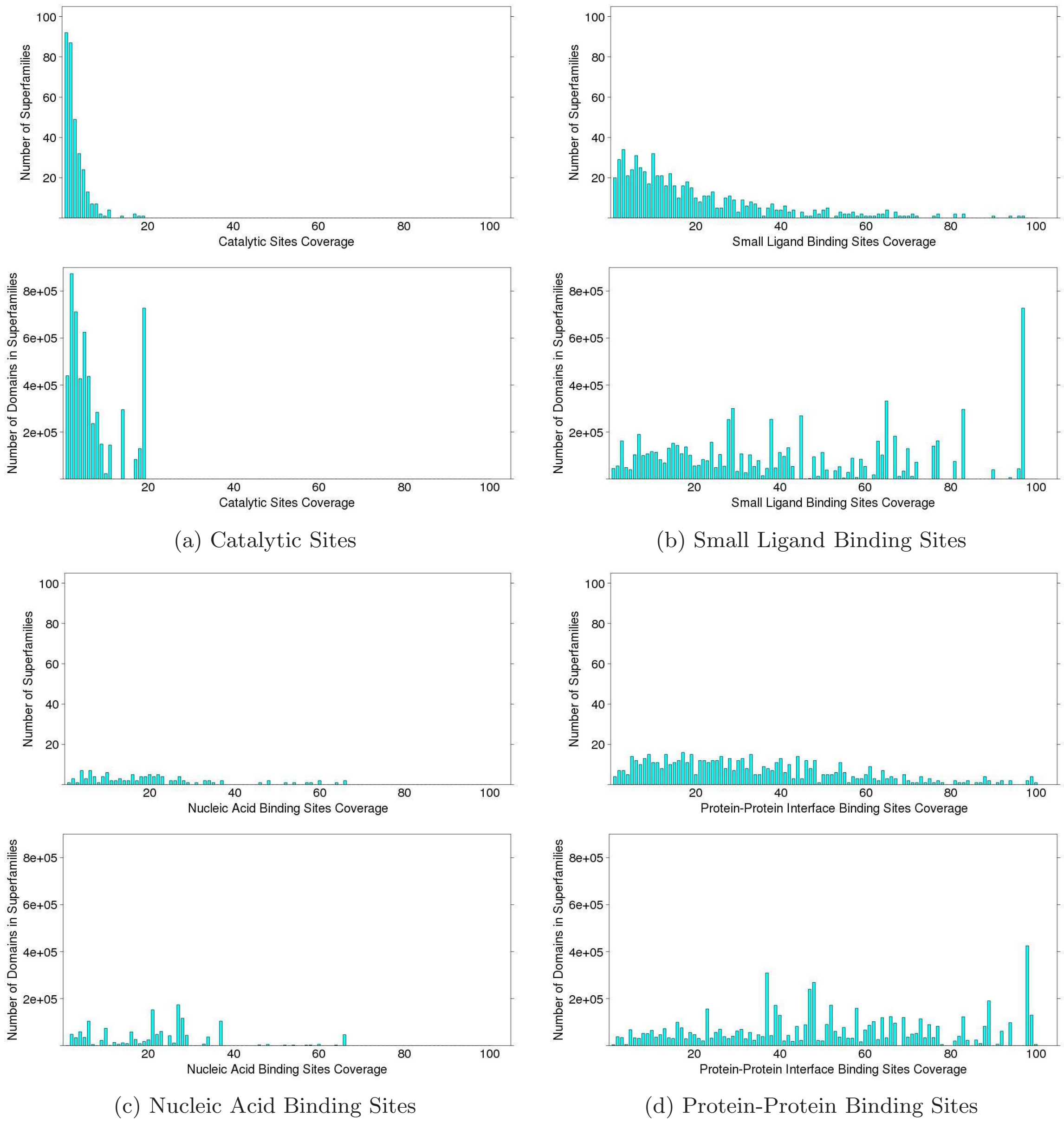

(a) Catalytic Sites

(b) Small Ligand Binding Sites

(c) Nucleic Acid Binding Sites

(d) Protein-Protein Binding Sites

Figure A.14: Functional site coverage, superfamily abundance and domain sequence abundance within superfamilies. On the X-axis, functional site coverage (defined in Figure 2) has been binned in one percentage bins, i.e 0-1%, 1-2%, 2-3% and so on. The number of superfamilies on the upper Y-axis is measured as the number of superfamilies with a functional site coverage that falls within a given bin. The number of domains in the superfamilies on the lower Y-axis is measured as the number of domain sequences within the superfamilies measured on the upper Y-axis.

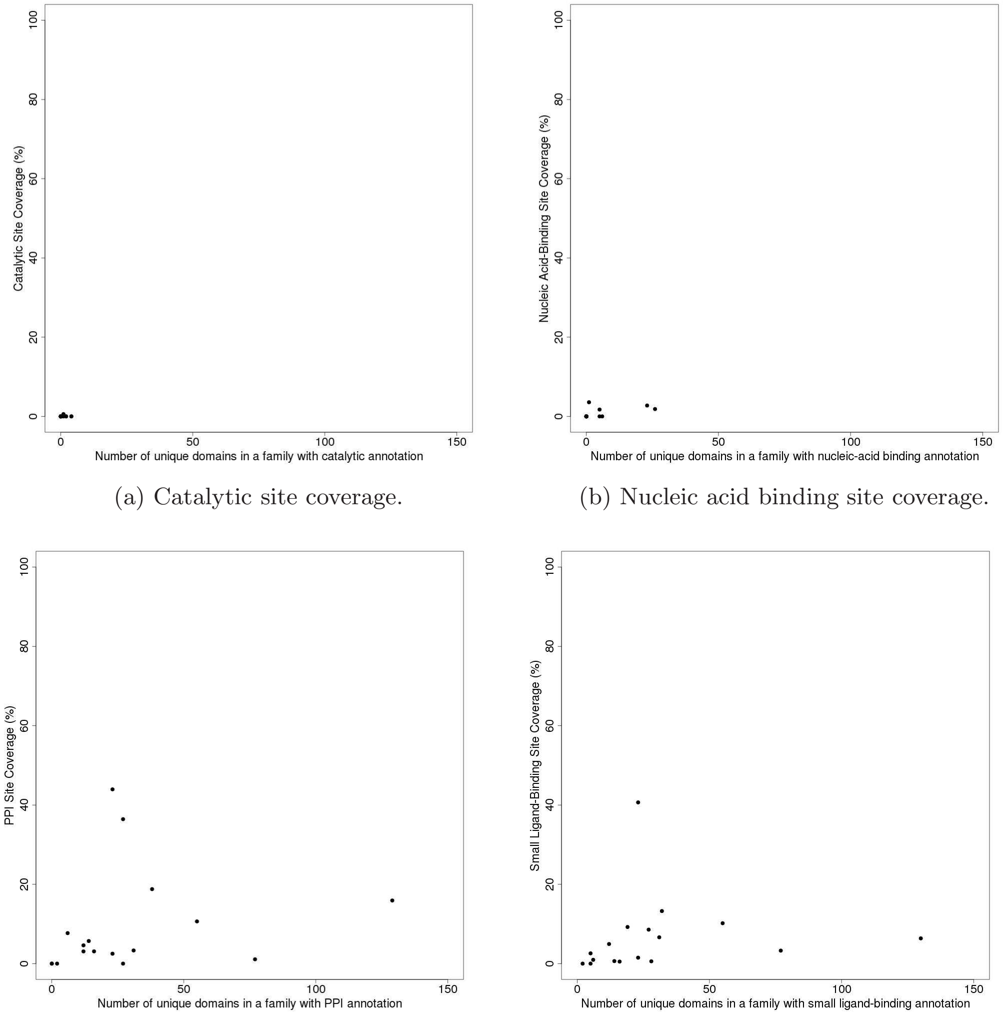

(a) Catalytic site coverage.

(b) Nucleic acid binding site coverage.

(c) Protein-protein binding site coverage.

(d) Small ligand binding site coverage.

Figure A.15: Functional site coverage versus the number of family domains with functional site annotation. Each dot represents a FineFam functional family within the HUPs superfamily (3.40.50.620).

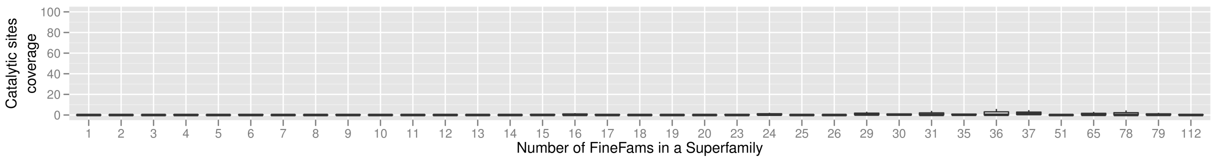

(a) Catalytic site coverage.

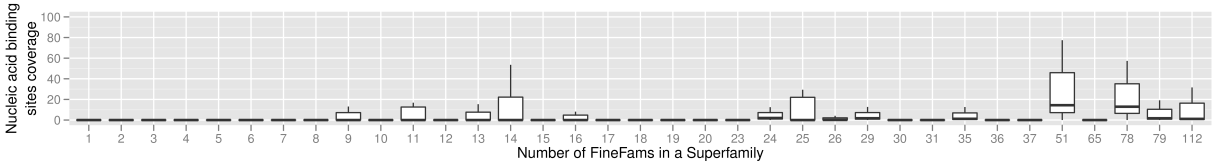

(b) Nucleic acid binding site coverage.

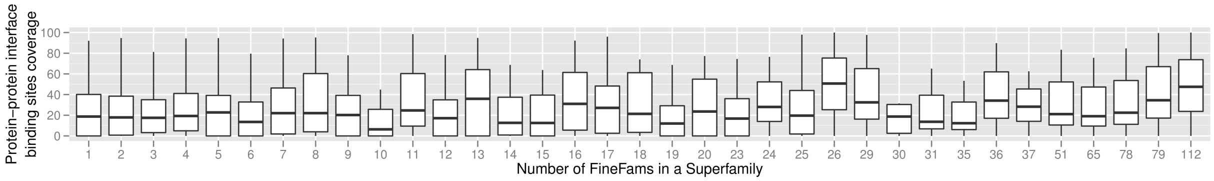

(c) Protein-protein binding site coverage.

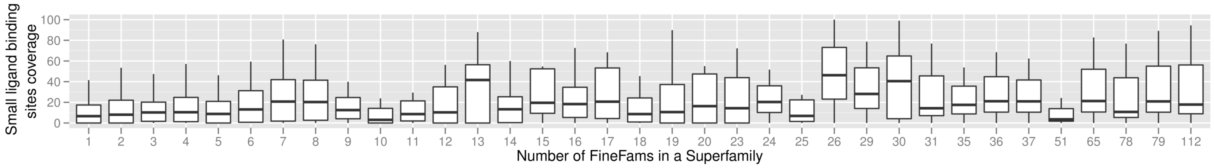

(d) Small ligand binding site coverage.

Figure A.16: Median functional site coverage for FineFams within a superfamily versus superfamily diversity, measured by the number of FineFam functional families. Each box-plot represents pooled data for those superfamilies with a given number of FineFam functional families. The bottom of a box represents the lower (first) quartile, or 25% of the data points. The line inside the box represents the median, or the second quartile, containing 50% of the data points. The top of the box represents the upper (third) quartile, or 75% of the data points. The upper whisker extends from the upper quartile to the highest data point that is within 1.5 x inter-quartile range of the upper quartile.

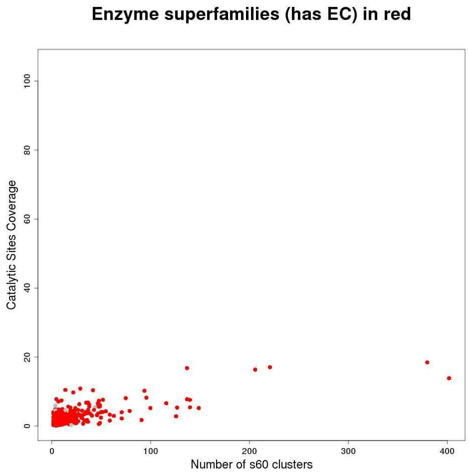

(a) Catalytic site coverage.

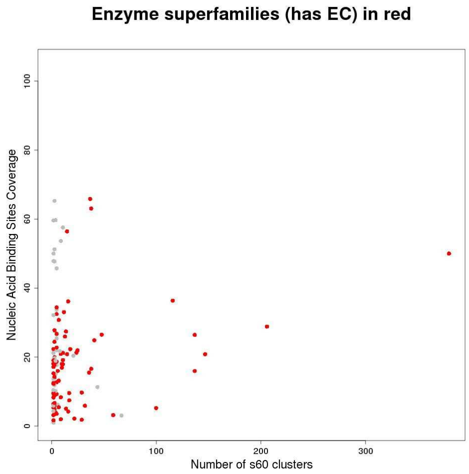

(b) Nucleic acid binding site coverage.

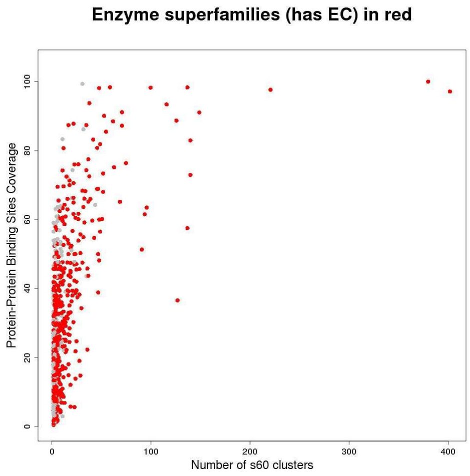

(c) Protein-protein binding site coverage.

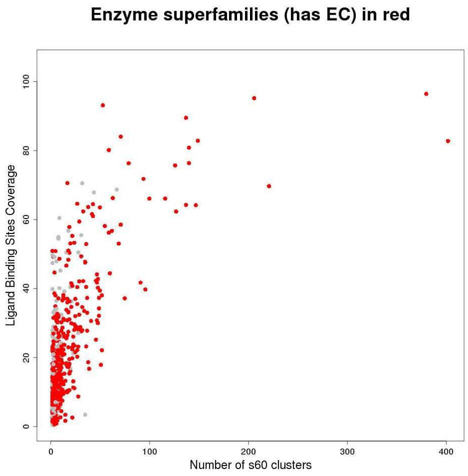

(d) Small ligand binding site coverage.

Figure A.17: Functional site coverage versus superfamily diversity, with the enzymatic superfamilies, i.e. those with EC numbers, shown in red.

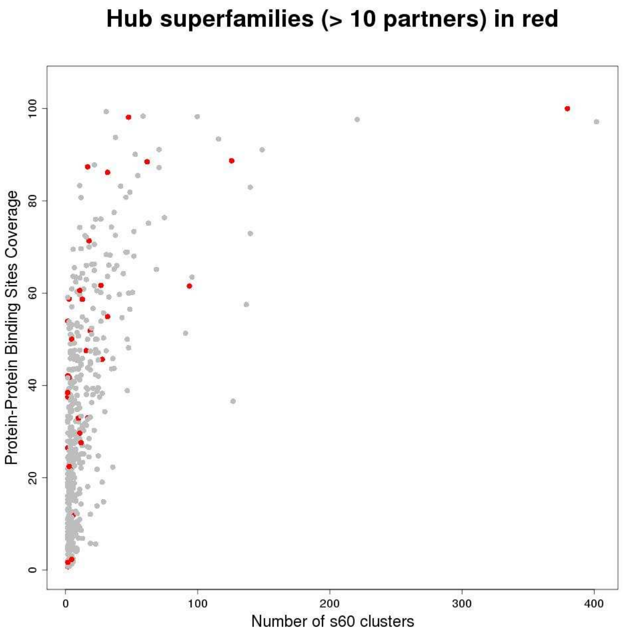

Figure A.18: Protein-Protein binding site coverage versus superfamily diversity. Each dot represents a superfamily. Red dots represent superfamilies where at least one member comes from a protein that interacts with more than 10 partners.

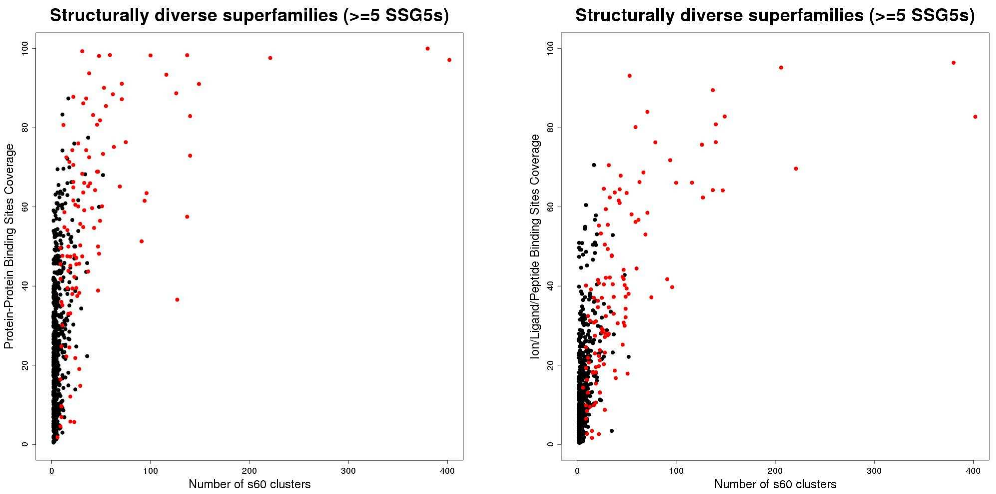

(a) Protein-protein interface coverage

(b) Small ligand binding site coverage

Figure A.19: Functional site coverage versus superfamily diversity. Structurally diverse superfamilies are shown in red, i.e. those with at least five structural clusters, where a cutoff of 5Å was used to generate the clusters.

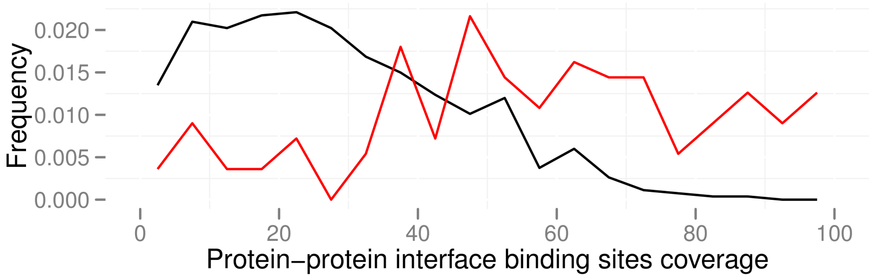

(a) Protein-protein interface binding sites coverage

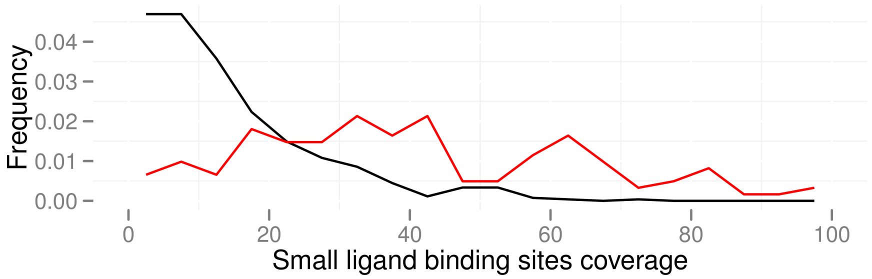

(b) Small ligand binding site coverage

Figure A.20: Proportional frequency versus functional site type coverage. The black line represents superfamilies with less than five structural clusters, and the red line represents superfamilies with at least five structural clusters. For each plot, a Wilcoxon Rank-Sum test found that there is a significant difference between the distributions of superfamilies that are not structurally divergent and the superfamilies that are. A p-value of less than 2.2 x 10^{-16} was calculated in both cases and the functional site coverage values in the structurally diverse superfamilies were shown to be significantly larger.

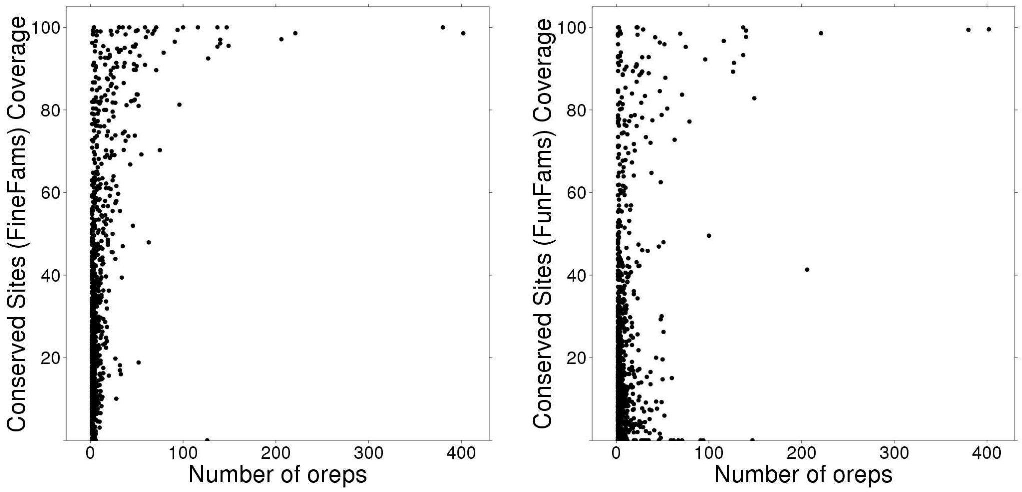

(a) FineFams.

(b) FunFams.

Figure A.21: Conserved site coverage versus functional family diversity.

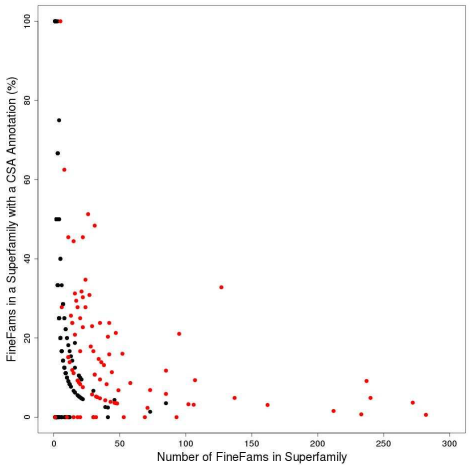

(a) Catalytic site coverage.

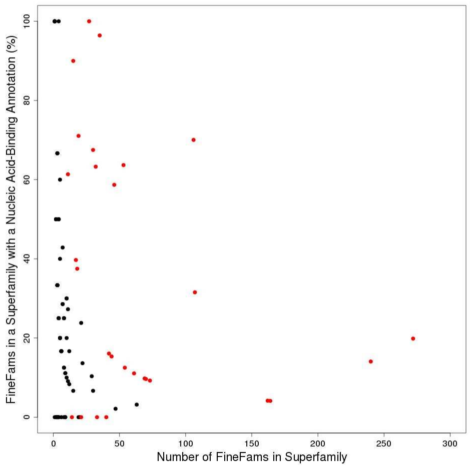

(b) Nucleic acid binding site coverage.

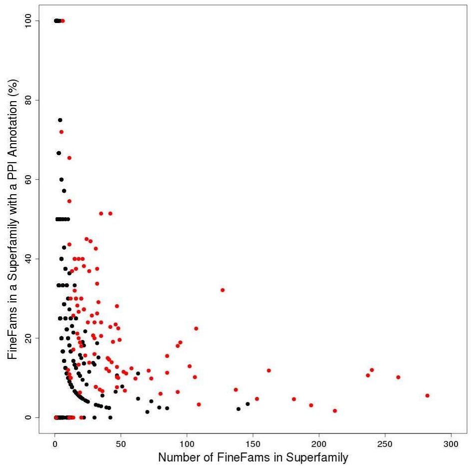

(c) Protein-protein binding site coverage.

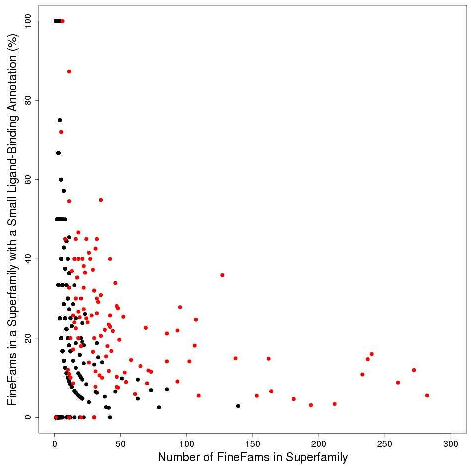

(d) Small ligand binding site coverage.

Figure A.22: The proportion of FineFams with an experimentally-derived annotation of a given functional type versus functional family diversity. Each dot represents a superfamily. Superfamilies with at least five structural clusters generated with a cutoff of 5Å are shown in red.
